# Quantification of spatial pharmacogene expression heterogeneity in breast tumors

**DOI:** 10.1002/cnr2.1686

**Published:** 2022-07-30

**Authors:** Nicholas R. Powell, Rebecca M. Silvola, John S. Howard, Sunil Badve, Todd C. Skaar, Joseph Ipe

**Affiliations:** ^1^ Department of Medicine, Division of Clinical Pharmacology Indiana University School of Medicine Indianapolis Indiana USA; ^2^ Department of Pathology and Laboratory Medicine Emory University School of Medicine Atlanta Georgia USA

**Keywords:** chemotherapy, pharmacogene, resistance, spatial, transcriptomics

## Abstract

**Background:**

Chemotherapeutic drug concentrations vary across different regions of tumors and this is thought to be involved in development of chemotherapy resistance. Insufficient drug delivery to some regions of the tumor may be due to spatial differences in expression of genes involved in the disposition, transport, and detoxification of drugs (pharmacogenes). Therefore, in this study, we analyzed the spatial expression of 286 pharmacogenes in six breast cancer tissues using the recently developed Visium spatial transcriptomics platform to (1) determine if these pharmacogenes are expressed heterogeneously across tumor tissue and (2) to determine which pharmacogenes have the most spatial expression heterogeneity.

**Methods and Results:**

The spatial transcriptomics technology sequences the transcriptome of 55 um diameter barcoded sections (spots) across a tissue sample. We analyzed spatial gene expression profiles of four biobank‐sourced breast tumor samples in addition to two breast tumor sample datasets from 10× Genomics. We define heterogeneity as the interquartile range of read counts. Collectively, we identified 8887 spots in tumor regions, 3814 in stroma, 44 in lymphocytes, and 116 in normal regions based on pathologist annotation of the tissues. We showed statistically significant differences in expression of pharmacogenes in tumor regions compared to surrounding non‐tumor regions. We also observed that the most heterogeneously expressed genes within tumor regions were involved in reactive oxygen species (ROS) handling and detoxification mechanisms. *GPX4, GSTP1, MGST3, SOD1, CYP4Z1, CYB5R3, GSTK1*, and *NAT1* showed the most heterogeneous expression within tumor regions.

**Conclusions:**

The heterogeneous expression of these pharmacogenes may have important implications for cancer therapy due to their ability to impact drug distribution and efficacy throughout the tumor. Our results suggest that chemoresistance caused by expression of *GPX4, GSTP1, MGST3*, and *SOD1* may be intrinsic, not acquired, since the heterogeneity is not specific to chemotherapy‐treated samples or cell type. Additionally, we identified candidate chemoresistance pharmacogenes that can be further tested through focused follow‐up studies.

## 1 BACKGROUND

Heterogeneity is one of the hallmarks of cancer^1^ and is associated with resistance to treatment, poor prognosis, and treatment failure.^2^, ^3^, ^4^ Intratumor heterogeneity exists at multiple levels including genetic, epigenetic, metabolic, and transcriptional.^5^, ^6^, ^7^, ^8^, ^9^, ^10^ Spatial transcriptomic heterogeneity, that is, a wide range in gene expression between neighboring regions of a tissue, can lead to phenotypic variability in traits such as cellular morphology,^11^ growth,^12^ metabolism,^13^ immune function,^14^ angiogenicity,^15^ reactive oxygen handling,^16^, ^17^ and solute transport.^18^ These phenotypic changes that result from transcriptionally distinct regions of the tumor (due to spatial heterogeneity) are significant because they are subsequently able to confer resistance to anti‐cancer chemotherapies.^19^

Success of systemic anti‐cancer chemotherapy is dependent upon the ability to deliver cytotoxic concentrations of the drug to all cells within a tumor. Drug concentrations vary across different regions of tumors^20^ and is thought to be involved in development of resistance.^21^ Insufficient drug delivery to some regions of the tumor may be due to spatial differences in expression of genes involved in the disposition and transport of drugs (pharmacogenes). Drug transporters such as the solute‐carrier protein (*SLC*) and the ATP‐binding cassette (*ABC*) family are membrane proteins that transport a wide variety of molecules, including chemotherapeutic agents, and have been linked with multidrug resistance in cancers.^22^, ^23^ This mechanism of resistance is thought to involve survival of certain tumor cells with strong drug efflux mechanisms,^24^, ^25^, ^26^, ^27^ which seed resistant cancer cells that clonally expand and regenerate the tumor after chemotherapy treatment.^28^

Transporters are not the only mechanism of chemotherapeutic resistance. For example, several chemotherapeutic agents alter the redox homeostasis of cancer cells by elevating levels of reactive oxygen species (ROS) and inducing ROS mediated cell injury.^29^, ^30^ Aberrant expression of ROS handling enzymes, including detoxifying enzymes such as superoxide dismutase (*SOD*)^31^ and glutathione peroxidase (*GPX*)^32^ can also promote development of regions across the tumors that are resistant to chemotherapeutic agents.

Spatial expression of other pharmacogenes involved in the pharmacokinetics (PK) of chemotherapeutic agents, like the cytochrome P450 (*CYP*) enzymes, could also be involved in spatial differences in tumor drug concentrations. Therefore, in this study, we analyzed the spatial expression of 286 pharmacogenes in 6 breast cancer tissues using the recently developed Visium spatial transcriptomics platform^33^ to determine if these pharmacogenes are expressed heterogeneously across tumors and to determine which pharmacogenes have the most spatial expression heterogeneity, as defined by interquartile range of read counts. The long‐term goal of this research is to better understand whether and how spatial heterogeneity in pharmacogene expression impacts chemotherapy resistance. This study is the next step toward this goal and is designed to identify candidate chemoresistance pharmacogenes that have the most variation in expression from region to region (1) across combined samples and within tumor or tumor‐adjacent breast tissue, and (2) between samples in tumor regions only. More specifically, the purpose of this study is to enable future studies to focus on a few candidate chemoresistance genes (narrowed down from 286 pharmacogenes) that can be perturbed in model systems of tumorigenesis and tested for their contribution to tumor sensitivity and resistance development to a single chemotherapeutic agent.

## 2 METHODS

### 2.1 Tissue collection

Tissues were obtained from the Indiana Biobank under an approved IRB protocol at Indiana University. Consent for research and publication of de‐identified research information was provided by subjects of the Indiana Biobank. Surgically resected breast tumors were weighed and divided into ~150 mg tissue pieces that were flash frozen in liquid nitrogen and placed in a cryovial. The tissues were bio‐banked and stored in liquid phase of nitrogen until experimental use.

### 2.2 Cryosectioning

A cryomold was placed in an ethanol‐dry ice slurry. Tissue was transferred from the cryovial on dry ice to the cryomold using pre‐cooled forceps. The pre‐cooled Optimum Cutting Temperature (OCT) compound was added to the cryomold, completely covering the tissue, and was allowed to freeze for approximately 2 min. The frozen tissue block was removed and placed on a cryotome chuck using OCT and immediately transferred to a pre‐chilled cryotome chamber (Leica CM3050 S). Once frozen, the cryotome chuck was placed on the stage and the tissues were cryosectioned at a thickness of 14–16 μm. The chamber temperature was maintained at −20°C and the specimen head was maintained at −28 to −32°C. Tissue sections were placed within the frames of a pre‐chilled Visium Spatial slide. The slide was transferred to a prechilled slide box and stored on dry ice.

### 2.3 Visium spatial tissue optimization and library preparation

Visium Spatial Tissue Optimization was done as per manufacturer's instructions using a breast tumor tissue sample sectioned at 16 μm. Based on the tissue optimization, the optimal permeabilization time was determined to be 12 min. Imaging was done using a Keyence BZ‐X microscope. Visium Spatial library preparation was done as per manufacturer's instructions. H&E‐stained tissue images were captured at 10× magnification. Imaging time was 14 min per slide. cDNA and library preparation were done at the Center for Medical Genomics core at Indiana University School of Medicine.

### 2.4 Sequencing and analysis

300 pM dual‐indexed libraries were sequenced using Novaseq 6000 and a SP flow cell at 200 cycles per lane. Sample demultiplexing, image alignment, barcode processing, and gene counting was done using the Space Ranger pipeline (10× Genomics). Briefly, spaceranger mkfastq was used to wrap Illumina's bcl2fastq to demultiplex Visium‐prepared sequencing runs and to convert barcode and read data to FASTQ files. Spaceranger count was then used to combine the FASTQ file and optical H&E stained tissue image, generate feature‐spot matrices, determine clusters, and perform initial gene expression analysis. A Loupe Browser file was created for an interactive visualization functionality.

### 2.5 Data analysis

Broad descriptions of the data analysis are given here, and the scripts used to analyze the data are provided in the supplement. We used R (version 3.6.0) for all analyses. First, pathologist annotated cluster assignments (barcode annotation index) were downloaded from the Loupe Browser and read into R along with the full unique molecular identifier (UMI) read count matrix for each sample. Data was either normalized to (divided by) the total reads per spot or kept as is, depending on the analysis. Data that was normalized was done so similar to the transcripts‐per‐million (TPM) method,^34^ without the unnecessary multiplication by 1 million. Log transformation was not performed on read counts for certain analyses (as indicated in the results section) to yield a more intuitive interpretation of the data. Log2 transformation was performed for fold‐change analysis. For differential expression analysis, read counts that were zero in one group and nonzero in another were retained in the analysis as positive or negative “infinity,” and shown above or below the solid horizontal line in the respective figure. While it is not inappropriate to discard read counts of zero, we kept them since these values are indicative of low expression and therefore are more informative to retain (e.g., a gene with four reads is likely more highly expressed than a gene with zero reads). Pharmacogenes were filtered based on a list of 298 pharmacogenes as described by www.pharmaadme.org, resulting in 286 pharmacogenes that were present in the read count matrix. The pharmacogene list is based on input from seven major pharmaceutical companies as to which genes perform or regulate drug metabolism or transport. Spots were categorized by sample or by annotation as needed for each analysis. Interquartile range of read counts (75th–25th percentile) was used to measure heterogeneity because it best characterizes the spread (variability) of data without being too heavily influenced by outlier values. Additionally, it allowed better visual representation than standard deviation or coefficient of variation. Spatial serial correlation (autocorrelation) was not used since our aims were focused on the spread of expression values, not the correlation of expression between neighboring spots.

To define which genes were related to reactive oxygen species handling (ROS), we used MGI's Batch Query (http://www.informatics.jax.org/batch/summary) to categorize pharmacogenes corresponding to ROS GO terms using key words “oxidative,” “stress,” “oxygen,” and “reactive,” followed by removal of CYP and ABC genes, to yield a final list of 35 “ROS genes” (corresponding to 27 ROS GO terms that can be found in Table S1).

### 2.6 GO pathway analysis

To determine which cellular pathways are associated with the 66 most heterogeneously expressed pharmacogenes across all 6 samples comprising our dataset, we used the PANTHER Gene List Analysis Statistical overrepresentation test (http://pantherdb.org/) to identify the top 15 significantly overrepresented GO terms (Fisher's exact, Bonferroni *p*‐value <.05) respective to each of the three annotation sets. Panther's over‐representation analysis (ORA) approach fulfilled our aim of identifying the top pathways associated with our dataset's most heterogeneously expressed genes.

### 2.7 Statistical analysis

Most of the analyses were descriptive in nature, except for the differential expression analysis. Log2 fold change comparisons for UMI‐normalized unique‐UMI reads, for tumor regions (per sample) versus combined non‐tumor regions across all samples, utilized two‐sided Welch's *t*‐tests and are Bonferroni corrected (*p*‐values are multiplied by the number of statistical tests performed).

## 3 RESULTS

### 3.1 Summary of tumor tissues

We analyzed spatial gene expression profiles of four biobank‐sourced breast tumor samples in addition to two breast tumor sample datasets from 10× Genomics. The patient demographics and disease characteristics of the biobank samples are listed in Table S2.

Spatial gene expression patterns were generated from a total of 13 600 spots across the six tissues with 27 542 genes detected. Figure S1 shows that pharmacogenes were evenly distributed across the relative expression range (Figure S1A) and that many of the CYP and SLC genes were generally expressed at lower levels (Figure S1B). Pharmacogenes involved in ROS handling (ROS genes) and the ABC transporters are generally seen toward the top half of relative expression among the pharmacogenes.

A pathologist's annotation (S.B.) of the H&E‐stained sections was used to classify spatially barcoded spots within the tissue as tumor, stroma, lymphocytes, normal, or mixed. A visual representation of the observed spatial heterogeneity in the expression of ABC transporters (as an example) within pathologist‐annotated tumor regions are shown in Figure S2.

Collectively, we identified 8887 spots in tumor regions, 3814 in stroma, 44 in lymphocytes, and 116 in normal regions. The remaining spots, ~5% of the total, were considered mixed regions that were primarily stroma with presence of tumor cells and were not included in the analysis.

### 3.2 Pharmacogene expression in tissues

We interrogated the spatial expression of 286 pharmacogenes across six tissue samples. Out of the 286 pharmacogenes, 259 were expressed in at least one sample and 214 were expressed in all six tissues.

**FIGURE 1:**
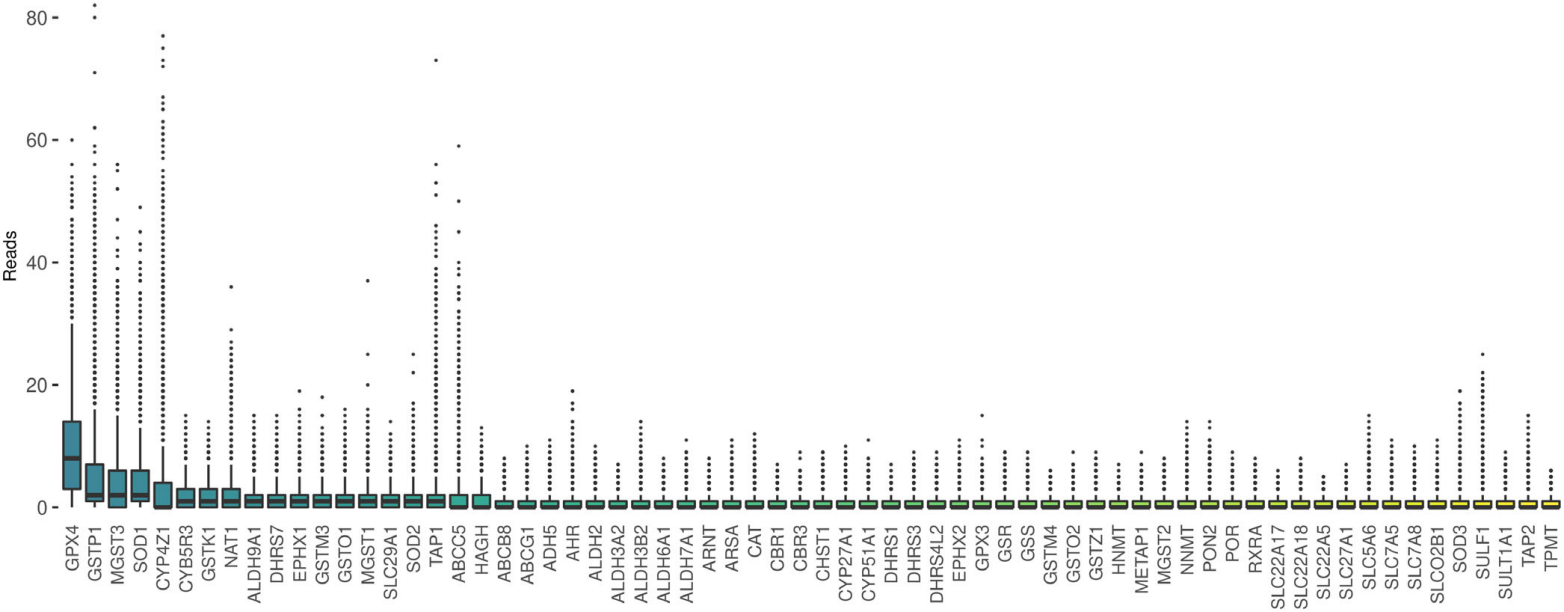
Boxplot of all pharmacogenes, across all samples, in tumor regions only, with an interquartile range greater than zero. Each data point on the *y*‐axis is the number of unique‐UMI reads from a single barcoded spot. The genes on the *X*‐axis are sorted by interquartile range in descending order

The interquartile range of gene expression across spatially barcoded spots was used as a measure of heterogeneous gene expression. We calculated the interquartile range of gene expression across pathologist‐annotated tumor spots in two ways; within each sample individually and combined across all tissue samples. Sixty‐six genes were found to have an interquartile range greater than zero, specifically in tumor regions, across the six samples. Figure 1 shows the expression levels of these genes within tumor spots from all six tissue samples. *GPX4*, *GSTP1*, *MGST3*, *SOD1*, *CYP4Z1*, *CYB5R3*, *GSTK1*, and *NAT1* showed the most heterogeneous expression.

**FIGURE 2:**
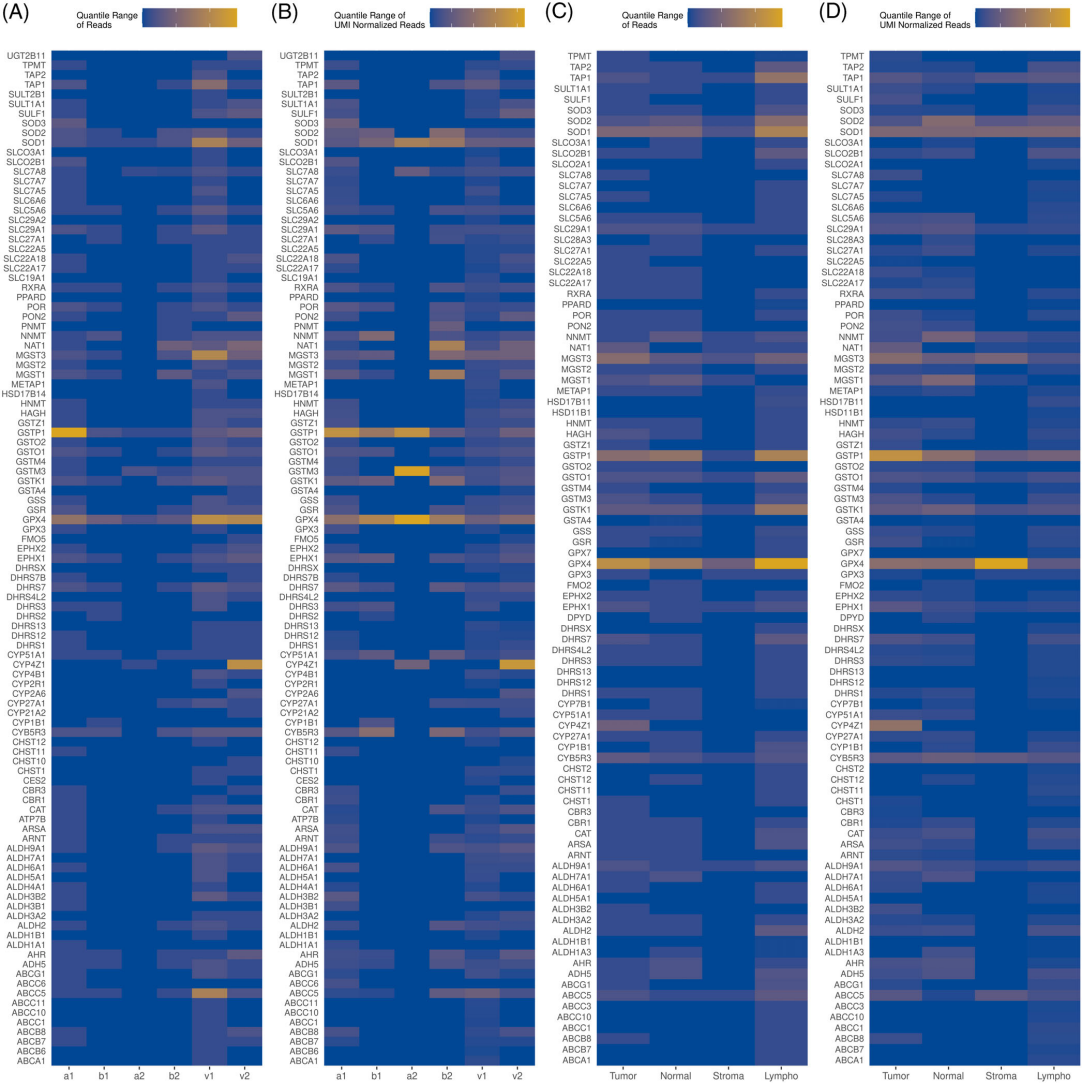
Intensity maps showing subsets of pharmacogenes with an interquartile range greater than 0 in any of the groups on the *x*‐axis. (A) Interquartile range of unique‐UMI reads for each gene for each sample in tumor spots. (B) Interquartile range of UMI‐normalized unique‐UMI reads for each gene for each sample in tumor spots. (C) Interquartile range of unique‐UMI reads for each gene for each annotated region. (D) Interquartile range of UMI‐normalized unique‐UMI reads for each gene for each annotated region

The heterogeneity of pharmacogene expression across just tumor spots within each of the six tissues is shown in Figure 2A. To account for possible variability in global transcriptional activity in each of the tumor spots, the interquartile range of expression was also determined after pharmacogene expression was normalized to total read counts from each spot (Figure 2B). We observe that *GPX4*, *GSTP1*, and *SOD1* are heterogeneously expressed across samples, whereas *CYP4Z1*, *GSTM3*, and *NAT1* were heterogeneously expressed in only some of the tissues. *CYB5R3* and *ABCC5* were heterogeneously expressed in all but one of the tissue samples.

We show that the expression of pharmacogenes is also heterogeneous in tumor‐associated stroma, lymphocytes, and adjacent normal regions. The interquartile range of pharmacogene expression (Figure 2C,D) in these regions demonstrates presence of heterogeneous gene expression in both tumor and tumor‐associated surrounding regions. For example, *GPX4*, *GSTP1*, *MGST3*, *SOD1*, and *CYB5R3* appear to be heterogeneously expressed despite the annotated cell type. Heterogeneous *CYP4Z1* expression was observed mainly in tumor regions, but this is likely because it was specifically higher in sample v2 which lacked any annotation of clearly normal regions.

**FIGURE 3:**
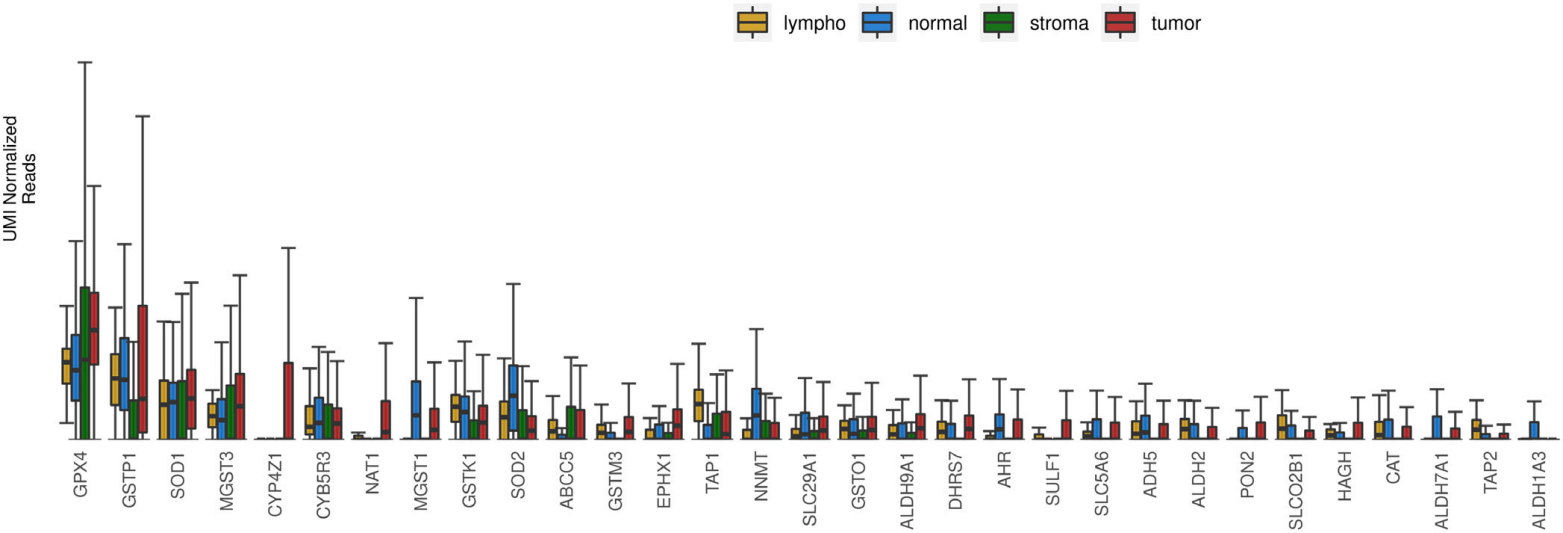
Boxplots of UMI‐normalized unique‐UMI reads for subsets of pharmacogenes with the top 31 largest interquartile ranges. Groups are divided into tumor (tumor + DCIS + cellular tumor + desmoplastic tumor), normal, lymphocytes, and stroma. *Y*‐axis scale is arbitrary, as this figure is intended to show relative differences. DCIS, ductal carcinoma in situ

The most highly heterogeneously expressed pharmacogenes and their observed expression in tumor, tumor‐associated stroma, lymphocytes, and normal tissues are shown in Figure 3. Figure S3 shows increased heterogeneity in some genes across tumor regions compared to the composite of other (non‐tumor) regions.

**FIGURE 4:**
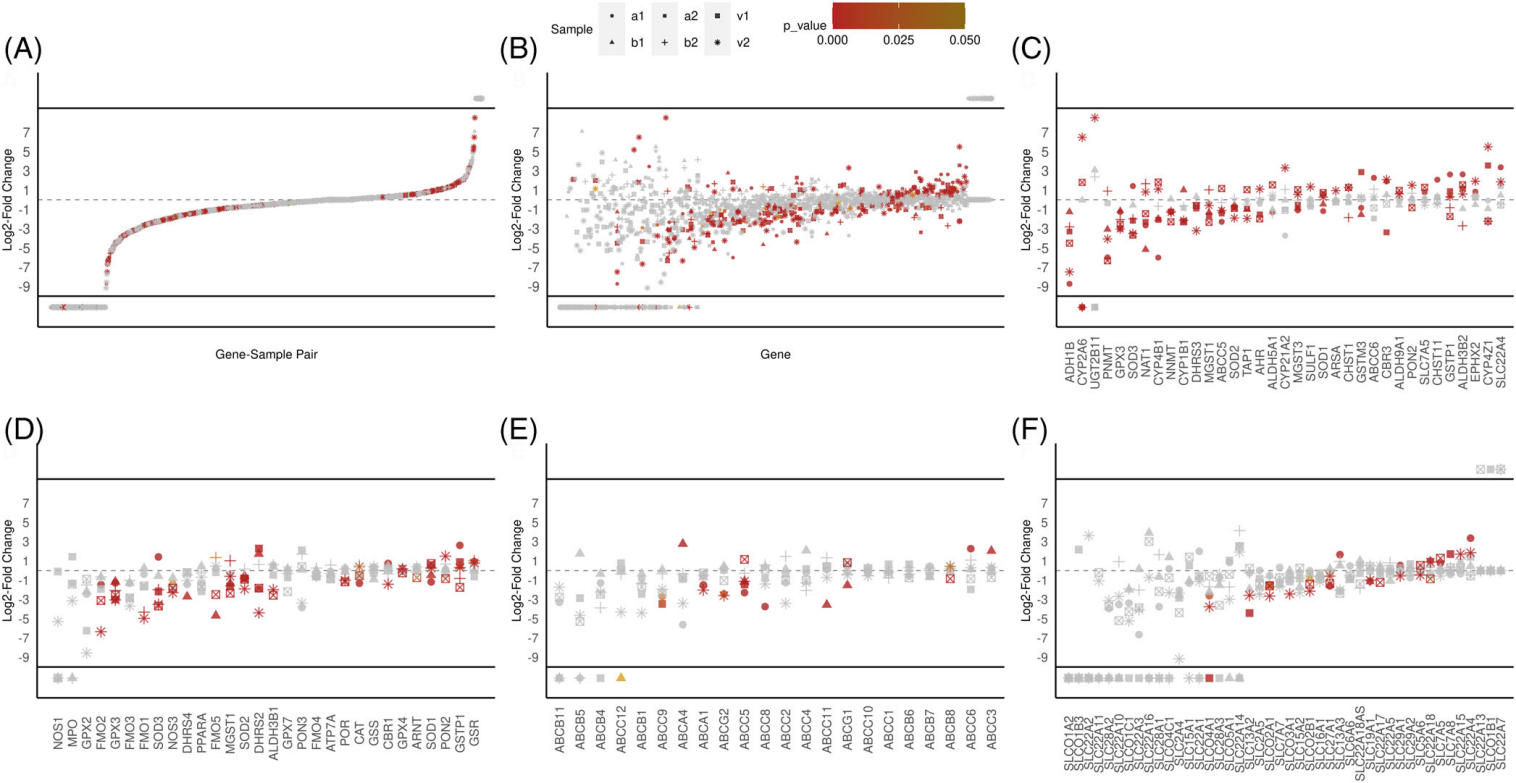
Log2 fold change comparisons for UMI‐normalized unique‐UMI reads, for tumor regions (per sample) versus combined non‐tumor regions across all samples. The *X*‐axis is in ascending order by average fold change per group. *p*‐values are based two‐sided Welch's *t*‐tests, are Bonferroni corrected, and are colored by intensity. Shapes denote which sample the fold change is based on. Values above and below the black bars represent divide‐by‐zero values of infinity or negative infinity; these values were kept because one of the groups in the comparison had an average of zero expression while the other group had non‐zero expression, indicating a potentially significant differential expression. (A) S‐plot showing the distribution for each gene‐sample combination. (B) Global comparison by pharmacogene. (C) Subset of 35 genes with the lowest *p*‐values. (D) Subset of genes involved in reactive oxygen species handling. (E) ABC transporter subset. (F) SLC transporters subset

In addition to heterogeneous expression, we observed statistically significant differences in the overall expression of pharmacogenes in tumor regions compared to adjacent regions. Figure 4A illustrates differential expression of pharmacogenes between tumor regions and adjacent non‐tumor regions. We noticed pharmacogenes tend to be downregulated in tumor regions as can be seen in Figure 4B, however there were still many significantly upregulated genes. Figure 4C shows the top 35 most differentially expressed genes, with *ADH1B* and *GPX3* being significantly downregulated in all six tissue samples. Figure 4D,E,F show subsets of differential expression for ROS genes, ABC transporter genes, and SLC transporter genes, respectively. These subsets generally do not show obvious differences in ROS or transporter gene expression associated with tumor pathology (except, perhaps, for *GPX3*). Samples a1 (paclitaxel and cyclophosphamide) and b1 (anastrazole and capecitabine) were post‐chemotherapy treated and showed significantly upregulated transporters in *ABCA4* (b1), *ABCC6* (a1), and *ABCC3* (b1). This study was not designed to test the effect of treatment on transporter expression‐mediated survival and selection, however, so these results should be viewed conservatively.

### 3.3 Biological processes altered by top hits

We conducted a GO pathway analysis to identify the GO terms that are significantly overrepresented by the 66 pharmacogenes with an interquartile range above zero in tumor regions. The 45 most significant GO terms (Bonferroni‐corrected *p*‐value <.05) associated with the 66 heterogeneously expressed pharmacogenes are listed in Table S3. We found a predominance of genes involved in reactive oxygen species handling in addition to drug and metabolite transport.

## 4 DISCUSSION

We analyzed the spatial expression patterns of pharmacogenes in six human breast tumor samples (including tumor and adjacent tissue) and found pharmacogene expression to be heterogeneous within tumor regions. We also showed statistically significant differences in expression of pharmacogenes in tumor regions compared to surrounding non‐tumor regions. We observed that the most heterogeneously expressed genes were involved in ROS handling and detoxification mechanisms. The heterogeneous expression of these pharmacogenes may have important implications for cancer therapy due to their ability to impact drug distribution and efficacy throughout the tumor.

The Visium Spatial Transcriptomics platform measures the expression of the whole transcriptome within intact fresh‐frozen tissue sections while simultaneously preserving the spatial context of gene expression. The technology relies on spatially barcoded poly‐T capture probes that hybridize with mRNA. Due to this non‐specific mRNA capture mechanism, highly expressed genes may be captured more frequently than genes expressed at relatively lower levels, limiting sensitivity. Among all six tissue specimens, 214 pharmacogenes were detected, albeit many at lower levels (below 20 unique reads) compared to the most highly expressed genes. While the relative expression of pharmacogenes across different histologic regions were comparable across all six tissue samples, larger numbers of uniquely barcoded reads were reported in samples with higher sequencing depth. Future development of capture probes specifically designed for pharmacogenes may improve the sensitivity of their detection. This limitation of low read counts was also problematic for reliably correlating the expression of one gene to another. For future studies intending to conduct linear regressions between genes using this technology, it would be ideal to optimize the methodology to maximize read depth.

Heterogeneity was defined as the interquartile range of unique‐UMI reads measured across each UMI‐barcoded pharmacogene for every spatial spot. Gene expression in each spot may not be uniform; for example, spots within stromal tissue sections will have markedly lower gene expression when compared to spots within tumor regions. To account for such variability in gene expression across the entire tissue, we normalized the read counts to the total number of UMIs (total unique reads) detected in each spot. We have presented the data both with and without such normalization because the process of normalization may undermine some of the observed heterogeneity in pharmacogenes. For example, overexpression of other cancer‐related genes relative to a given pharmacogene in a tumor region may result in a higher total UMI count leading to a masking effect caused by normalization.

Our study evaluated spatial heterogeneity in tumor pharmacogene expression but did not evaluate the downstream impact of this heterogeneity. Therefore, our results should be regarded as hypothesis‐generating and not as direct evidence behind chemoresistance mechanisms. However, the literature supports the hypothesis that spatial heterogeneity in tumor pharmacogene expression contributes to chemotherapeutic resistance mechanisms. For example, certain drug and metabolite transporters, such as those included in the multidrug resistance protein (MRP) group of the large ABC family, are known to play a role in chemoresistance when overexpressed as they contribute to the efflux of chemotherapeutic compounds from the cell.^35^ One of these transporters, ABCC5, was among our most heterogeneously expressed pharmacogenes. ABCC5 transports cyclic nucleotides, including the metabolites of 5‐fluorouracil (5‐FU), a common anticancer agent used in both breast cancer and colon cancer treatment. ABCC5‐transfected cells have been shown to have a nearly 9‐fold increase in 5‐FU resistance.^35^ Another transporter in this same family also found to be heterogeneously expressed is ABCB8; it transports compounds from the mitochondria to the cytosol. One study showed inhibition of ABCB8 with shRNA resulted in increased doxorubicin‐induced mitochondrial DNA damage.^36^ Combined with literature knowledge that these genes can affect 5‐FU (or capecitabine) and doxorubicin resistance, respectively, we are now able to hypothesize that large degrees of expression heterogeneity (e.g., in ABCC5 and ABCB8) are more likely to seed the clonal expansion of post‐capecitabine/doxorubicin surviving resistant cells. Therefore, it is now possible to test this hypothesis, for example, in a sufficiently large sample size to determine if ABCB8 is overexpressed in human doxorubicin‐resistant tumor samples versus treatment‐naïve tumor samples. Additionally, further application of spatial transcriptomics technology to in vivo doxorubicin‐tumor response models would allow visualization of the clonal expansion and emergence of ABCB8‐mediated resistance over the time‐course of doxorubicin tumor treatment.

Several of the pharmacogenes with the highest heterogeneity (e.g., *GPX4, GSTP1, MGST3*, and *SOD1*) are also known to impact drug response. Glutathione peroxidases (GPX) are enzymes that protect cells from oxidative stress by catalyzing the reduction of peroxides. GPX4 has been shown to be critical for survival of lapatinib‐resistant cancer cells, but not lapatinib‐naïve cancer cells.^32^ In colorectal cancer, increased GPX3 expression resulted in increased resistance to oxaliplatin and cisplatin.^37^ This evidence, combined with our detection of spatial heterogeneity in *GPX3* and *GPX4* expression within tumors, could explain why certain cells survive platinum‐agent or lapatinib treatment, leading to resistant tumors.

*GSTP1* was also highly heterogeneously expressed in our data. Enzymes in the glutathione s‐transferase (GST) family catalyze the conjugation of polar glutathione groups that enhance systemic elimination of chemotherapeutic agents and toxic metabolites. GST activity has been shown to be inducible by treatment with vincristine, doxorubicin, or topotecan.^38^ When GSTP1 enzymatic activity is impaired, as is the case with the rs1695 missense variant,^39^ platinum‐based chemotherapy‐induced granulocytopenia was shown to be more common in a meta‐analysis of 12 case control trials.^40^ Additionally, *GSTP1* expression was found to be higher in adriamycin‐resistant cells, and higher *GSTP1* expression was also found in breast cancer tissues from subjects with progressive/stable disease versus those with partial/complete response. Interestingly, this finding was true for tissue collected before and after anthracycline/taxane treatment, indicating intrinsic mechanisms of resistance. These data indicate that spatial differences in *GSTP1* expression could be involved in chemoresistance and seeding of resistant tumor cells following chemotherapy treatment.

*MGST3* was another highly heterogeneously expressed gene in our data. This enzyme is involved in immune function by catalyzing the conjugation of reduced glutathione and leukotriene A4, producing leukotriene C4. Overexpression of *MGST3* was found in cisplatin resistant lung adenocarcinoma cells compared to non‐resistant progenitor cells, and when *MGST3* expression was increased (via antagonism with its microRNA regulator, mir‐432‐5p) the progenitor cells demonstrated increased survival to cisplatin treatment.^41^ Although it is unclear if this relates to immune modulation, these data and other similar studies^42^, ^43^ indicate that spatial differences in *MGST3* expression could be involved in chemoresistance and seeding of resistant tumor cells following chemotherapy treatment.

Another highly heterogeneously expressed gene in our data was superoxide dismutase 1 (*SOD1*). SOD1 eliminates damaging superoxide radicals by converting them to less toxic molecular oxygen and hydrogen peroxide. SOD activity counteracts superoxide‐induced autophagy^44^ and inhibition of *SOD1* with siRNA or small molecule inhibitors results in increased cisplatin sensitivity in ovarian cancer cells.^45^, ^46^ These studies combined with our results suggest spatial differences in *SOD1* expression could be involved in chemoresistance and seeding of resistant tumor cells following chemotherapy treatment.

We found that reactive oxygen species handling (by gene families like GPX, GSTP, and SOD), is a pathway that is overrepresented among the pharmacogenes that were heterogeneously expressed in our data. The over‐representation analysis (ORA) approach identified the top pathways associated with our dataset's most heterogeneously expressed genes.

Our study also assessed differential pharmacogene expression between tumor versus tumor‐adjacent regions. This analysis was secondary to the goal of characterizing pharmacogene expression heterogeneity, but does provide some insight into the effect of pharmacogene regulation in tumor regions. Interestingly, *ADH1B* and *GPX3* were downregulated in all six samples. As discussed earlier, GPX3 may play a role in resistance to platinum agents, and while it is not clear how, tumor‐induced downregulation could somehow be involved. A potentially more relevant conclusion is that tumor regions do not appear to consistently induce expression of pharmacogenes that could predispose to chemotherapeutic resistance.

We acknowledge some additional limitations to our study. First, we have a small sample size and more information will likely be gained from bigger studies. Second, the lower expressed genes may contain additional variability that we did not observe due to the low read numbers. Third, the resolution between spots is not fine enough to capture single cells, so there may be some overlap in cell types. Lastly, we were not able to identify somatic mutations that may account for variability in pharmacogene expression.

## 5 CONCLUSIONS

Our data show substantial heterogeneity in the expression of many pharmacogenes across areas of the breast tumors. These results provide more quantitative measurements of expression heterogeneity across spatial regions of tumors. Our results provide more detail on the current understanding of chemoresistance development by providing evidence that there is heterogeneity in the expression of these chemoresistance genes across tumor sub‐regions. Our results suggest that chemoresistance caused by GPX4, GSTP1, MGST3, and SOD1 may be intrinsic, not acquired, since the heterogeneity is not specific to chemotherapy‐treated samples or cell type. Additionally, we demonstrate the utility of spatial transcriptomics to identify candidate chemoresistance pharmacogenes that can now be further tested through focused follow‐up studies.

## AUTHOR CONTRIBUTIONS

**Nicholas R. Powell:** Conceptualization (supporting); data curation (lead); formal analysis (lead); methodology (supporting); writing – original draft (equal); writing – review and editing (equal). **Rebecca M. Silvola:** Conceptualization (supporting); data curation (supporting); formal analysis (equal); methodology (supporting); writing – original draft (supporting); writing – review and editing (supporting). **John S. Howard:** Conceptualization (supporting); data curation (supporting); formal analysis (supporting); methodology (supporting); writing – original draft (supporting); writing – review and editing (supporting). **Sunil Badve:** Conceptualization (supporting); data curation (equal); formal analysis (supporting); methodology (supporting); writing – original draft (supporting); writing – review and editing (supporting). **Todd C. Skaar:** Conceptualization (equal); data curation (supporting); formal analysis (supporting); funding acquisition (equal); methodology (supporting); writing – original draft (supporting); writing – review and editing (supporting). **Joseph Ipe:** Conceptualization (lead); data curation (equal); formal analysis (equal); funding acquisition (equal); methodology (lead); writing – original draft (equal); writing – review and editing (equal).

## FUNDING INFORMATION

This work was supported by NIH‐NIGMS grants: T32GM008425 (N.R.P.), and T32GM842528 (R.M.S.), the Vera Bradley Foundation for Breast Cancer Research (J.I.), and the Indiana University Grand Challenge Precision Health Initiative (J.I.).

## CONFLICT OF INTEREST

Joseph Ipe began working at 10× Genomics after this study was completed, and while no conflict of interest existed during the study, this statement is provided for full disclosure. None of the other authors have potential conflicts of interest related to this work.

## ETHICS STATEMENT

Tissues were obtained from the Indiana Biobank under the approved IRB protocol (number 1803676586) at Indiana University.

## CONSENT FOR PUBLICATION

Consent for research and publication of de‐identified research information was provided by subjects of the Indiana Biobank.

## Supporting information


**Appendix S1**: Supporting Information.

**Figure S1**: Comparison of expression levels of pharmacogenes. Expression is plotted on the *y*‐axis as the log10 of mRNA reads per sample normalized to the total number of spots for that sample for each gene on the *x*‐axis. Order is determined by average of the relative expression in each group. (A) All genes in gray versus pharmacogenes in red. (B) Pharmacogenes divided by category.
**Figure S2**: Tissue images showing the barcoded dots where combined unique‐UMI reads of the ABC transporters are greater than 0. Color intensity represents number of reads, and circles with black outlines denote the regions that were pathologist annotated as tumor.
**Figure S3**: Boxplots of UMI‐normalized unique‐UMI reads for subsets of pharmacogenes with the largest interquartile ranges. Groups divided into tumor (tumor + DCIS + cellular tumor + desmoplastic tumor) and non‐tumor (normal + lymphocytes + stroma), showing the gene subset based on interquartile ranges greater than 0. *Y*‐axis scale is arbitrary, as this figure is intended to show relative differences.

**Table S1**: ROS GO terms and GO IDs. We filtered GO terms using the following key words; “oxidative” (*n* = 9), “oxygen” (*n* = 43), and “reactive” (*n* = 6). We then removed 34 terms that lacked clear ROS function to arrive at 27 unique terms corresponding to 35 ROS pharmacogenes.
**Table S2**: Patient demographics for samples obtained from the Indiana Biobank.
**Table S3**: The 45 most significant GO terms (Bonferroni‐corrected *p*‐value <.05) overrepresented by the 66 heterogeneously expressed pharmacogenes. Within the PANTHER web tool, we ran statistical overrepresentation tests (Fisher's exact and Bonferroni correction) for the three annotation sets using all human genes in the database as the reference list.

## Data Availability

Data files are available upon reasonable request from the corresponding author.
