# Supplementary material for: Quantification of spatial pharmacogene expression heterogeneity in breast tumors
Source: Cancer Rep (Hoboken). 2022 Jul 30;6(1):e1686. doi: 10.1002/cnr2.1686 (PMC9875649; doi:10.1002/cnr2.1686)
Supplement: Supplementary file 1 — Appendix S1: Supporting Information. [file CNR2-6-e1686-s003.docx]

Some of the R pipeline was adapted from snippets of code provided by 10x genomics for secondary analysis (<https://support.10xgenomics.com/spatial-gene-expression/software/pipelines/latest/rkit>).

############## install any of these packages first, as needed ###############################

setwd("/User/Visium")

library(ggplot2)

library(Matrix)

library(rjson)

library(cowplot)

library(RColorBrewer)

library(grid)

library(readbitmap)

library(Seurat)

library(dplyr)

library(hdf5r)

library(data.table)

library(ggpubr)

library(ggforce)

library(scales)

library(Ipaper) #only need for boxplots without outliers

library(ComplexHeatmap)

############## define sample names ################################################

sample_names <- c("a1", "b1","a2","b2","v1","v2")

############## define paths #############################################################

image_paths_hires <- c("/User/Visium/outs.a1/spatial/tissue_hires_image.png",

"/User/Visium/outs.b1/spatial/tissue_hires_image.png",

"/User/Visium/outs.a2/spatial/tissue_hires_image.png",

"/User/Visium/outs.b2/spatial/tissue_hires_image.png",

"/User/Visium/outs.v1/spatial/tissue_hires_image.png",

"/User/Visium/outs.v2/spatial/tissue_hires_image.png",

"/User/Visium/outs.d1/spatial/tissue_hires_image.png")

scalefactor_paths <- c("/User/Visium/outs.a1/spatial/scalefactors_json.json",

"/User/Visium/outs.b1/spatial/scalefactors_json.json",

"/User/Visium/outs.a2/spatial/scalefactors_json.json",

"/User/Visium/outs.b2/spatial/scalefactors_json.json",

"/User/Visium/outs.v1/spatial/scalefactors_json.json",

"/User/Visium/outs.v2/spatial/scalefactors_json.json",

"/User/Visium/outs.d1/spatial/scalefactors_json.json")

tissue_paths <- c("/User/Visium/outs.a1/spatial/tissue_positions_list.csv",

"/User/Visium/outs.b1/spatial/tissue_positions_list.csv",

"/User/Visium/outs.a2/spatial/tissue_positions_list.csv",

"/User/Visium/outs.b2/spatial/tissue_positions_list.csv",

"/User/Visium/outs.v1/spatial/tissue_positions_list.csv",

"/User/Visium/outs.v2/spatial/tissue_positions_list.csv",

"/User/Visium/outs.d1/spatial/tissue_positions_list.csv")

cluster_paths_pathol <- c("/User/Visium/outs.a1/analysis/clustering/graphclust/clusters_pathol.csv",

"/User/Visium/outs.b1/analysis/clustering/graphclust/clusters_pathol.csv",

"/User/Visium/outs.a2/analysis/clustering/graphclust/clusters_pathol.csv",

"/User/Visium/outs.b2/analysis/clustering/graphclust/clusters_pathol.csv",

"/User/Visium/outs.v1/analysis/clustering/graphclust/clusters_pathol.csv",

"/User/Visium/outs.v2/analysis/clustering/graphclust/clusters_pathol.csv",

"/User/Visium/outs.d1/analysis/clustering/graphclust/clusters_pathol.csv")

# matrix_paths <- c("/User/Visium/outs.a1/filtered_feature_bc_matrix.h5",

# "/User/Visium/outs.b1/filtered_feature_bc_matrix.h5",

# "/User/Visium/outs.a2/filtered_feature_bc_matrix.h5",

# "/User/Visium/outs.b2/filtered_feature_bc_matrix.h5",

# "/User/Visium/outs.v1/filtered_feature_bc_matrix.h5",

# "/User/Visium/outs.v2/filtered_feature_bc_matrix.h5",

# "/User/Visium/outs.d1/filtered_feature_bc_matrix.h5")

############## read in pathol defined clusters #########################################

clusters_pathol <- list()

for (i in 1:length(sample_names)) {

clusters_pathol[[i]] <- read.csv(cluster_paths_pathol[i])

}

############## read in the Full matrix and barcodes and genes ###############################################

matrix <- list()

for (i in 1:length(sample_names)) {

matrix_dir = paste0("/User/Visium/outs.",sample_names[i],"/filtered_feature_bc_matrix/")

barcode.path <- paste0(matrix_dir, "barcodes.tsv.gz")

features.path <- paste0(matrix_dir, "features.tsv.gz")

matrix.path <- paste0(matrix_dir, "matrix.mtx.gz")

matrix[[i]] <- t(readMM(file = matrix.path))

feature.names = read.delim(features.path,

header = FALSE,

stringsAsFactors = FALSE)

barcode.names = read.delim(barcode.path,

header = FALSE,

stringsAsFactors = FALSE)

rownames(matrix[[i]]) = barcode.names$V1

colnames(matrix[[i]]) = feature.names$V2

}

############## UMI normalization ###################################################

matrix_UMInorm <- list()

for (i in 1:length(sample_names)) {

UMIsum <- Matrix::rowSums(matrix[[i]])

matrix_UMInorm[[i]] <- matrix[[i]]/UMIsum

}

### Figure 1 ###################

############## Filtering matrix down to just tumor + DCIS + In situ regions #################

tumor_barcodes <- list()

for(i in 1:length(sample_names)){

tumor_barcodes[[i]] <- subset(clusters_pathol[[i]],clusters_pathol[[i]][["Pathol"]]=="Tumor" |

clusters_pathol[[i]][["Pathol"]]=="tumor" |

clusters_pathol[[i]][["Pathol"]]=="?DCIS" |

clusters_pathol[[i]][["Pathol"]]=="In situ" |

clusters_pathol[[i]][["Pathol"]]=="Cellular tumor" |

clusters_pathol[[i]][["Pathol"]]=="desmoplastic tumor")

}

tumor_matrix <- list()

for (i in 1:length(sample_names)) {

tumor_matrix[[i]] <- subset.matrix(matrix[[i]],matrix[[i]]@Dimnames[[1]] %in% tumor_barcodes[[i]][["Barcode"]])

}

############## filter tumor_matrix to pharmacogenes ################################

pharmacogenes <- read.csv("/User/Visium/Transporters and Pharmacogenes (Expanded).csv", header=FALSE)

pharmacogenes <- as.vector(pharmacogenes$V2)

genes_in_data <- vector() #this figures out the genes that are in the tumor_matrix data

for (i in 1:length(sample_names)) {

genes_in_data <- append(genes_in_data,tumor_matrix[[i]]@Dimnames[[2]])

}

genes_in_data <- unique(genes_in_data)

pharm_in_data <- subset(pharmacogenes, pharmacogenes %in% genes_in_data) #this picks out the pharamcogenes from our list that are in the data

tumor_pharm_genes <- list() #this creates a list of tables for the tumor pharmacogenes

for (i in 1:length(sample_names)) {

tumor_pharm_genes[[i]] <- as.data.table(tumor_matrix[[i]])[, pharm_in_data, with=FALSE]

}

############## Prep for plotting ##########################################

tumor_pharm_combined <- data.frame() #combines samples

for (i in 1:length(sample_names)) {

y <- as.data.frame(tumor_pharm_genes[[i]])

y$sample <- sample_names[[i]]

tumor_pharm_combined <- rbind(tumor_pharm_combined,y)

}

z <- tumor_pharm_combined[-c(length(tumor_pharm_combined))]

z1 <- data.frame(colnames(z))

colnames(z1) <- "gene"

zz <- reshape2::melt(tumor_pharm_combined)

#for figure 1B

for(n in 1:length(z)){ #this allows to subset the list based on quantile range

z1[n,2] <- mean(z[,n])

z1[n,3] <- quantile(z[,n],probs=0.25)

z1[n,4] <- quantile(z[,n],probs=0.75)

z1[n,5] <- z1[n,4]-z1[n,3]

}

z2 <- subset(z1,z1$V5>0) #selects which genes to plot

pharm_in_data2 <- as.vector(z2$gene) #for figure 1B subset

# some functions

scale_fill_pharm <- function(...){

ggplot2:::manual_scale(

'fill',

values = setNames(c("goldenrod3","firebrick3","dodgerblue3","green4","darkslategrey"),

c('a1','b1','a2','b2','v1')),

...

)

}

percentile_function <- function(a){

-(quantile(a,probs=0.75,na.rm = T)-quantile(a,probs=0.25,na.rm = T))

}

percentile_function2 <- function(b){

-((quantile(b,probs=0.75,na.rm = T)-quantile(b,probs=0.25,na.rm = T))+(median(b,na.rm=T))/100)

}

############ PLOT Unused ##########################

# box1a <- ggplot(data=zz,mapping=aes(x=variable,y=value))+

# scale_fill_viridis_d()+

# geom_boxplot2()+theme(

# axis.text.x = element_blank(),

# axis.title.y = element_text(size=8),

# axis.title.x = element_blank(),

# axis.ticks.x = element_blank(),

# panel.grid.minor = element_blank(),

# panel.background = element_blank(),

# legend.position = "none")+

# ylab("Reads")

# #box1a

# ggsave("Figure1A.tiff",plot = box1a,device = "tiff",dpi=200,units='in',height=4,width = 30)

############ PLOT 1 ##########################

zzz <- zz[zz$variable %in% pharm_in_data2,]

box1b <- ggplot(data=zzz,mapping=aes(x=reorder(variable,value,FUN = percentile_function2),

y=value,fill=reorder(variable,value,FUN = percentile_function2)))+

geom_boxplot(outlier.size = 0.1,alpha=0.9)+theme(

axis.text.x = element_text(angle=90,size=8,vjust=.5,hjust = 1),

axis.title.y = element_text(size=8),

axis.title.x = element_blank(),

axis.ticks.x = element_blank(),

panel.grid.minor = element_blank(),

panel.background = element_blank(),

legend.position = "none")+

scale_fill_viridis_d(begin = 0.4)+

scale_y_continuous(expand = c(0,1))+

ylab("Reads")

#box1b

### Figure 2C ###################

############## filter Full matrix to pharmacogenes ################################

pharmacogenes <- read.csv("/User/Visium/Transporters and Pharmacogenes (Expanded).csv", header=FALSE)

pharmacogenes <- as.vector(pharmacogenes$V2)

genes_in_data <- vector() #this figures out the genes that are in the matrix data

for (i in 1:length(sample_names)) {

genes_in_data <- append(genes_in_data,matrix[[i]]@Dimnames[[2]])

}

genes_in_data <- unique(genes_in_data)

pharm_in_data <- subset(pharmacogenes,

pharmacogenes %in% genes_in_data) #this picks out the pharamcogenes from our list that are in the data

pharm_matrix <- list() #this creates a matrix for the pharmacogenes

for (i in 1:length(sample_names)) {

pharm_matrix[[i]] <- matrix[[i]][, pharm_in_data, with=FALSE]

}

############## Filtering pharm_matrix down to Tumor #################

tumor_pharm_barcodes <- list()

for(i in 1:length(sample_names)){

tumor_pharm_barcodes[[i]] <- subset(clusters_pathol[[i]],clusters_pathol[[i]][["Pathol"]]=="Tumor" |

clusters_pathol[[i]][["Pathol"]]=="tumor" |

clusters_pathol[[i]][["Pathol"]]=="?DCIS" |

clusters_pathol[[i]][["Pathol"]]=="In situ" |

clusters_pathol[[i]][["Pathol"]]=="Cellular tumor" |

clusters_pathol[[i]][["Pathol"]]=="desmoplastic tumor")

}

tumor_pharm_matrix <- list()

for (i in 1:length(sample_names)) {

tumor_pharm_matrix[[i]] <- subset.matrix(pharm_matrix[[i]],

pharm_matrix[[i]]@Dimnames[[1]] %in% tumor_pharm_barcodes[[i]][["Barcode"]])

}

tumor_pharm_genes <- list() #this creates a list of tables

for (i in 1:length(sample_names)) {

tumor_pharm_genes[[i]] <- as.data.table(tumor_pharm_matrix[[i]])

}

tumor_pharm_combined <- data.frame() #combines samples

for (i in 1:length(sample_names)) {

y <- as.data.frame(tumor_pharm_genes[[i]])

skip_to_next <- FALSE

tryCatch({y$sample <- sample_names[[i]]},error=function(e){ skip_to_next <<- TRUE})

if(skip_to_next) { next }

tumor_pharm_combined <- rbind(tumor_pharm_combined,y)

}

z <- tumor_pharm_combined[-c(length(tumor_pharm_combined))]

z1 <- data.frame(colnames(z))

colnames(z1) <- "gene"

for(n in 1:length(z)){ #gets quantile range for each gene-region pair

z1[n,2] <- mean(z[,n])

z1[n,3] <- quantile(z[,n],probs=0.25)

z1[n,4] <- quantile(z[,n],probs=0.75)

z1[n,5] <- z1[n,4]-z1[n,3]

}

z1 <- z1[c(1,5)]

colnames(z1)[2] <- "Tumor"

tumor <- z1

############## Filtering pharm_matrix down to Normal #################

normal_pharm_barcodes <- list()

for(i in 1:length(sample_names)){

normal_pharm_barcodes[[i]] <- subset(clusters_pathol[[i]],clusters_pathol[[i]][["Pathol"]]=="Normal" |

clusters_pathol[[i]][["Pathol"]]=="normal")

}

normal_pharm_matrix <- list()

for (i in 1:length(sample_names)) {

normal_pharm_matrix[[i]] <- subset.matrix(pharm_matrix[[i]],

pharm_matrix[[i]]@Dimnames[[1]] %in% normal_pharm_barcodes[[i]][["Barcode"]])

}

normal_pharm_genes <- list() #this creates a list of tables

for (i in 1:length(sample_names)) {

normal_pharm_genes[[i]] <- as.data.table(normal_pharm_matrix[[i]])

}

normal_pharm_combined <- data.frame() #combines samples

for (i in 1:length(sample_names)) {

y <- as.data.frame(normal_pharm_genes[[i]])

skip_to_next <- FALSE

tryCatch({y$sample <- sample_names[[i]]},error=function(e){ skip_to_next <<- TRUE})

if(skip_to_next) { next }

normal_pharm_combined <- rbind(normal_pharm_combined,y)

}

za <- normal_pharm_combined[-c(length(normal_pharm_combined))]

za1 <- data.frame(colnames(za))

colnames(za1) <- "gene"

for(n in 1:length(za)){ #gets quantile range for each gene-region pair

za1[n,2] <- mean(za[,n])

za1[n,3] <- quantile(za[,n],probs=0.25)

za1[n,4] <- quantile(za[,n],probs=0.75)

za1[n,5] <- za1[n,4]-za1[n,3]

}

za1 <- za1[c(1,5)]

colnames(za1)[2] <- "Normal"

normal <- za1

############## Filtering pharm_matrix down to Lympho #################

lympho_pharm_barcodes <- list()

for(i in 1:length(sample_names)){

lympho_pharm_barcodes[[i]] <- subset(clusters_pathol[[i]],clusters_pathol[[i]][["Pathol"]]=="lympho" |

clusters_pathol[[i]][["Pathol"]]=="Lympho")

}

lympho_pharm_matrix <- list()

for (i in 1:length(sample_names)) {

lympho_pharm_matrix[[i]] <- subset.matrix(pharm_matrix[[i]],

pharm_matrix[[i]]@Dimnames[[1]] %in% lympho_pharm_barcodes[[i]][["Barcode"]])

}

lympho_pharm_genes <- list() #this creates a list of tables

for (i in 1:length(sample_names)) {

lympho_pharm_genes[[i]] <- as.data.table(lympho_pharm_matrix[[i]])

}

lympho_pharm_combined <- data.frame() #combines samples

for (i in 1:length(sample_names)) {

y <- as.data.frame(lympho_pharm_genes[[i]])

skip_to_next <- FALSE

tryCatch({y$sample <- sample_names[[i]]},error=function(e){ skip_to_next <<- TRUE})

if(skip_to_next) { next }

lympho_pharm_combined <- rbind(lympho_pharm_combined,y)

}

zb <- lympho_pharm_combined[-c(length(lympho_pharm_combined))]

zb1 <- data.frame(colnames(zb))

colnames(zb1) <- "gene"

for(n in 1:length(zb)){ #gets quantile range for each gene-region pair

zb1[n,2] <- mean(zb[,n])

zb1[n,3] <- quantile(zb[,n],probs=0.25)

zb1[n,4] <- quantile(zb[,n],probs=0.75)

zb1[n,5] <- zb1[n,4]-zb1[n,3]

}

zb1 <- zb1[c(1,5)]

colnames(zb1)[2] <- "Lympho"

lympho <- zb1

############## Filtering pharm_matrix down to Stroma #################

stroma_pharm_barcodes <- list()

for(i in 1:length(sample_names)){

stroma_pharm_barcodes[[i]] <- subset(clusters_pathol[[i]],clusters_pathol[[i]][["Pathol"]]=="stroma" |

clusters_pathol[[i]][["Pathol"]]=="Stroma")

}

stroma_pharm_matrix <- list()

for (i in 1:length(sample_names)) {

stroma_pharm_matrix[[i]] <- subset.matrix(pharm_matrix[[i]],

pharm_matrix[[i]]@Dimnames[[1]] %in% stroma_pharm_barcodes[[i]][["Barcode"]])

}

stroma_pharm_genes <- list() #this creates a list of tables

for (i in 1:length(sample_names)) {

stroma_pharm_genes[[i]] <- as.data.table(stroma_pharm_matrix[[i]])

}

stroma_pharm_combined <- data.frame() #combines samples

for (i in 1:length(sample_names)) {

y <- as.data.frame(stroma_pharm_genes[[i]])

skip_to_next <- FALSE

tryCatch({y$sample <- sample_names[[i]]},error=function(e){ skip_to_next <<- TRUE})

if(skip_to_next) { next }

stroma_pharm_combined <- rbind(stroma_pharm_combined,y)

}

zc <- stroma_pharm_combined[-c(length(stroma_pharm_combined))]

zc1 <- data.frame(colnames(zc))

colnames(zc1) <- "gene"

for(n in 1:length(zc)){ #gets quantile range for each gene-region pair

zc1[n,2] <- mean(zc[,n])

zc1[n,3] <- quantile(zc[,n],probs=0.25)

zc1[n,4] <- quantile(zc[,n],probs=0.75)

zc1[n,5] <- zc1[n,4]-zc1[n,3]

}

zc1 <- zc1[c(1,5)]

colnames(zc1)[2] <- "Stroma"

stroma <- zc1

############## merge regions #################

quant <- merge(tumor, normal, by="gene")

quant <- merge(quant,stroma,by="gene")

quant <- merge(quant,lympho,by="gene")

quant$add <- rowSums(quant[,2:5])

quant_subset <- subset(quant, quant$add>0)

quant_subset <- quant_subset[c(1:5)]

quant_subset_melt <- reshape2::melt(quant_subset)

############ PLOT 2C ################

heat2c <- ggplot(quant_subset_melt,aes(y=gene, x=variable,fill=value))+

geom_tile(width=1, height=1)+

theme(axis.text.y = element_text(size=8),

axis.title.x = element_blank(),

axis.ticks.y = element_blank(),

axis.title.y = element_blank(),

legend.title = element_text(size=8),

legend.text = element_blank())+

scale_fill_gradient(low = muted("dodgerblue4"),high = "goldenrod")+

#scale_fill_gradientn(colors=c("darkslategrey","firebrick4"))+

scale_x_discrete(expand = c(0,0))+labs(fill="Quantile Range\nof Reads")

#heat2c

#ggsave("Figure2C.tiff",plot=heat2c,device='tiff',dpi=400, units = 'mm',height = 250, width = 100)

### Figure 2D ###################

############## filter Full matrix to pharmacogenes ################################

pharmacogenes <- read.csv("/User/Visium/Transporters and Pharmacogenes (Expanded).csv", header=FALSE)

pharmacogenes <- as.vector(pharmacogenes$V2)

genes_in_data <- vector() #this figures out the genes that are in the matrix data

for (i in 1:length(sample_names)) {

genes_in_data <- append(genes_in_data,matrix[[i]]@Dimnames[[2]])

}

genes_in_data <- unique(genes_in_data)

pharm_in_data <- subset(pharmacogenes,

pharmacogenes %in% genes_in_data) #this picks out the pharamcogenes from our list that are in the data

pharm_matrix <- list() #this creates a matrix for the pharmacogenes

for (i in 1:length(sample_names)) {

pharm_matrix[[i]] <- matrix[[i]][, pharm_in_data, with=FALSE]

}

############## Filtering pharm_matrix down to Tumor #################

tumor_pharm_barcodes <- list()

for(i in 1:length(sample_names)){

tumor_pharm_barcodes[[i]] <- subset(clusters_pathol[[i]],clusters_pathol[[i]][["Pathol"]]=="Tumor" |

clusters_pathol[[i]][["Pathol"]]=="tumor" |

clusters_pathol[[i]][["Pathol"]]=="?DCIS" |

clusters_pathol[[i]][["Pathol"]]=="In situ" |

clusters_pathol[[i]][["Pathol"]]=="Cellular tumor" |

clusters_pathol[[i]][["Pathol"]]=="desmoplastic tumor")

}

tumor_pharm_matrix <- list()

for (i in 1:length(sample_names)) {

tumor_pharm_matrix[[i]] <- subset.matrix(pharm_matrix[[i]],

pharm_matrix[[i]]@Dimnames[[1]] %in% tumor_pharm_barcodes[[i]][["Barcode"]])

}

tumor_pharm_genes <- list() #this creates a list of tables

for (i in 1:length(sample_names)) {

tumor_pharm_genes[[i]] <- as.data.table(tumor_pharm_matrix[[i]])

}

############## divide into samples ###############

x <- list()

for(i in 1:length(sample_names)){

z <- as.data.frame(tumor_pharm_genes[[i]])

z1 <- data.frame(colnames(tumor_pharm_genes[[i]]))

colnames(z1) <- c("gene")

for(n in 1:nrow(z1)){ #gets quantile range for each gene-sample pair

z1[n,2] <- mean(z[,n])

z1[n,3] <- quantile(z[,n],probs=0.25)

z1[n,4] <- quantile(z[,n],probs=0.75)

z1[n,5] <- z1[n,4]-z1[n,3]

}

x[[i]] <- z1

}

############## merge with samples in columns #################

y <- x[[1]]["gene"]

for(n in 1:length(sample_names)){

y[,n+1] <- x[[n]]["V5"]

}

colnames(y)[2:(length(sample_names)+1)] <- sample_names

y$add <- rowSums(y[,2:(length(sample_names)+1)],na.rm = TRUE)

y_subset <- subset(y, y$add>0)

y_subset <- y_subset[c(1:(length(sample_names)+1))]

y_subset_melt <- reshape2::melt(y_subset)

############ PLOT 2D ################

heat2d <- ggplot(y_subset_melt,aes(y=gene, x=variable,fill=value))+

geom_tile(width=1, height=1)+

theme(axis.text.y = element_text(size=8),

axis.title.x = element_blank(),

axis.ticks.y = element_blank(),

axis.title.y = element_blank(),

legend.text = element_blank(),

legend.title = element_text(size=8))+

scale_fill_gradient(low = muted("dodgerblue4"),high = "goldenrod")+

scale_x_discrete(expand = c(0,0))+labs(fill="Quantile Range\nof Reads")

#heat2d

#ggsave("Figure2D.tiff",plot=heat2d,device='tiff',dpi=400, units = 'mm',height = 250, width = 100)

### Figure S2 and Figure 3 ###################

variable <- 0.0001

############## filter Full matrix_UMInorm to pharmacogenes ################################

pharmacogenes <- read.csv("/User/Visium/Transporters and Pharmacogenes (Expanded).csv", header=FALSE)

pharmacogenes <- as.vector(pharmacogenes$V2)

genes_in_data <- vector() #this figures out the genes that are in the matrix_UMInorm data

for (i in 1:length(sample_names)) {

genes_in_data <- append(genes_in_data,matrix_UMInorm[[i]]@Dimnames[[2]])

}

genes_in_data <- unique(genes_in_data)

pharm_in_data <- subset(pharmacogenes,

pharmacogenes %in% genes_in_data) #this picks out the pharamcogenes from our list that are in the data

pharm_matrix_UMInorm <- list() #this creates a matrix for the pharmacogenes

for (i in 1:length(sample_names)) {

pharm_matrix_UMInorm[[i]] <- matrix_UMInorm[[i]][, pharm_in_data, with=FALSE]

}

############## Filtering pharm_matrix_UMInorm down to just tumor + DCIS + In situ + other tumor regions #################

tumor_barcodes <- list()

for(i in 1:length(sample_names)){

tumor_barcodes[[i]] <- subset(clusters_pathol[[i]],clusters_pathol[[i]][["Pathol"]]=="Tumor" |

clusters_pathol[[i]][["Pathol"]]=="tumor" |

clusters_pathol[[i]][["Pathol"]]=="?DCIS" |

clusters_pathol[[i]][["Pathol"]]=="In situ" |

clusters_pathol[[i]][["Pathol"]]=="Cellular tumor" |

clusters_pathol[[i]][["Pathol"]]=="desmoplastic tumor")

}

tumor_pharm_matrix_UMInorm <- list()

for (i in 1:length(sample_names)) {

tumor_pharm_matrix_UMInorm[[i]] <- subset.matrix(pharm_matrix_UMInorm[[i]],pharm_matrix_UMInorm[[i]]@Dimnames[[1]] %in% tumor_barcodes[[i]][["Barcode"]])

}

tumor_pharm_genes <- list() #this creates a list of tables

for (i in 1:length(sample_names)) {

tumor_pharm_genes[[i]] <- as.data.table(tumor_pharm_matrix_UMInorm[[i]])

}

############## Prep for plotting

tumor_pharm_combined <- data.frame() #combines samples

for (i in 1:length(sample_names)) {

y <- as.data.frame(tumor_pharm_genes[[i]])

y$sample <- sample_names[[i]]

tumor_pharm_combined <- rbind(tumor_pharm_combined,y)

}

z_tumor <- tumor_pharm_combined[-c(length(tumor_pharm_combined))]

z_tumor1 <- data.frame(colnames(z_tumor))

colnames(z_tumor1) <- "gene"

zz_tumor <- reshape2::melt(tumor_pharm_combined)

for(n in 1:length(z_tumor)){ #this allows to subset the list based on quantile range

z_tumor1[n,2] <- mean(z_tumor[,n])

z_tumor1[n,3] <- quantile(z_tumor[,n],probs=0.25)

z_tumor1[n,4] <- quantile(z_tumor[,n],probs=0.75)

z_tumor1[n,5] <- z_tumor1[n,4]-z_tumor1[n,3]

}

z_tumor2 <- subset(z_tumor1,z_tumor1$V5>0)

tumor_pharm_in_data2 <- as.vector(z_tumor2$gene)

z_tumor3 <- subset(z_tumor1,z_tumor1$V5>variable)

tumor_pharm_in_data3 <- as.vector(z_tumor3$gene)

############## Filtering pharm_matrix_UMInorm down to just normal, lympho, and stroma regions #################

nontumor_barcodes <- list()

for(i in 1:length(sample_names)){

nontumor_barcodes[[i]] <- subset(clusters_pathol[[i]],clusters_pathol[[i]][["Pathol"]]=="Normal" |

clusters_pathol[[i]][["Pathol"]]=="normal" |

clusters_pathol[[i]][["Pathol"]]=="lympho" |

clusters_pathol[[i]][["Pathol"]]=="Lympho" |

clusters_pathol[[i]][["Pathol"]]=="stroma" |

clusters_pathol[[i]][["Pathol"]]=="Stroma")

}

nontumor_pharm_matrix_UMInorm <- list()

for (i in 1:length(sample_names)) {

nontumor_pharm_matrix_UMInorm[[i]] <- subset.matrix(pharm_matrix_UMInorm[[i]],pharm_matrix_UMInorm[[i]]@Dimnames[[1]] %in% nontumor_barcodes[[i]][["Barcode"]])

}

nontumor_pharm_genes <- list() #this creates a list of tables

for (i in 1:length(sample_names)) {

nontumor_pharm_genes[[i]] <- as.data.table(nontumor_pharm_matrix_UMInorm[[i]])

}

############## Prep for plotting

nontumor_pharm_combined <- data.frame() #combines samples

for (i in 1:length(sample_names)) {

skip_to_next <- FALSE

y <- as.data.frame(nontumor_pharm_genes[[i]])

tryCatch(y$sample <- sample_names[[i]],error=function(e) { skip_to_next <<- TRUE})

if(skip_to_next) { next }

nontumor_pharm_combined <- rbind(nontumor_pharm_combined,y)

}

z_nontumor <- nontumor_pharm_combined[-c(length(nontumor_pharm_combined))]

z_nontumor1 <- data.frame(colnames(z_nontumor))

colnames(z_nontumor1) <- "gene"

zz_nontumor <- reshape2::melt(nontumor_pharm_combined)

for(n in 1:length(z_nontumor)){ #this allows to subset the list based on quantile range

z_nontumor1[n,2] <- mean(z_nontumor[,n])

z_nontumor1[n,3] <- quantile(z_nontumor[,n],probs=0.25)

z_nontumor1[n,4] <- quantile(z_nontumor[,n],probs=0.75)

z_nontumor1[n,5] <- z_nontumor1[n,4]-z_nontumor1[n,3]

}

z_nontumor2 <- subset(z_nontumor1,z_nontumor1$V5>0)

nontumor_pharm_in_data2 <- as.vector(z_nontumor2$gene)

z_nontumor3 <- subset(z_nontumor1,z_nontumor1$V5>variable)

nontumor_pharm_in_data3 <- as.vector(z_nontumor3$gene)

############## Filtering pharm_matrix_UMInorm down to just normal regions #################

normal_barcodes <- list()

for(i in 1:length(sample_names)){

normal_barcodes[[i]] <- subset(clusters_pathol[[i]],clusters_pathol[[i]][["Pathol"]]=="Normal" |

clusters_pathol[[i]][["Pathol"]]=="normal")

}

normal_pharm_matrix_UMInorm <- list()

for (i in 1:length(sample_names)) {

normal_pharm_matrix_UMInorm[[i]] <- subset.matrix(pharm_matrix_UMInorm[[i]],pharm_matrix_UMInorm[[i]]@Dimnames[[1]] %in% normal_barcodes[[i]][["Barcode"]])

}

normal_pharm_genes <- list() #this creates a list of tables

for (i in 1:length(sample_names)) {

normal_pharm_genes[[i]] <- as.data.table(normal_pharm_matrix_UMInorm[[i]])

}

############## Prep for plotting

normal_pharm_combined <- data.frame() #combines samples

for (i in 1:length(sample_names)) {

skip_to_next <- FALSE

y <- as.data.frame(normal_pharm_genes[[i]])

tryCatch(y$sample <- sample_names[[i]],error=function(e) { skip_to_next <<- TRUE})

if(skip_to_next) { next }

normal_pharm_combined <- rbind(normal_pharm_combined,y)

}

z_normal <- normal_pharm_combined[-c(length(normal_pharm_combined))]

z_normal1 <- data.frame(colnames(z_normal))

colnames(z_normal1) <- "gene"

zz_normal <- reshape2::melt(normal_pharm_combined)

for(n in 1:length(z_normal)){ #this allows to subset the list based on quantile range

z_normal1[n,2] <- mean(z_normal[,n])

z_normal1[n,3] <- quantile(z_normal[,n],probs=0.25)

z_normal1[n,4] <- quantile(z_normal[,n],probs=0.75)

z_normal1[n,5] <- z_normal1[n,4]-z_normal1[n,3]

}

z_normal2 <- subset(z_normal1,z_normal1$V5>0)

normal_pharm_in_data2 <- as.vector(z_normal2$gene)

z_normal3 <- subset(z_normal1,z_normal1$V5>variable)

normal_pharm_in_data3 <- as.vector(z_normal3$gene)

############## Filtering pharm_matrix_UMInorm down to just stroma regions #################

stroma_barcodes <- list()

for(i in 1:length(sample_names)){

stroma_barcodes[[i]] <- subset(clusters_pathol[[i]],clusters_pathol[[i]][["Pathol"]]=="stroma" |

clusters_pathol[[i]][["Pathol"]]=="Stroma")

}

stroma_pharm_matrix_UMInorm <- list()

for (i in 1:length(sample_names)) {

stroma_pharm_matrix_UMInorm[[i]] <- subset.matrix(pharm_matrix_UMInorm[[i]],pharm_matrix_UMInorm[[i]]@Dimnames[[1]] %in% stroma_barcodes[[i]][["Barcode"]])

}

stroma_pharm_genes <- list() #this creates a list of tables

for (i in 1:length(sample_names)) {

stroma_pharm_genes[[i]] <- as.data.table(stroma_pharm_matrix_UMInorm[[i]])

}

############## Prep for plotting

stroma_pharm_combined <- data.frame() #combines samples

for (i in 1:length(sample_names)) {

skip_to_next <- FALSE

y <- as.data.frame(stroma_pharm_genes[[i]])

tryCatch(y$sample <- sample_names[[i]],error=function(e) { skip_to_next <<- TRUE})

if(skip_to_next) { next }

stroma_pharm_combined <- rbind(stroma_pharm_combined,y)

}

z_stroma <- stroma_pharm_combined[-c(length(stroma_pharm_combined))]

z_stroma1 <- data.frame(colnames(z_stroma))

colnames(z_stroma1) <- "gene"

zz_stroma <- reshape2::melt(stroma_pharm_combined)

for(n in 1:length(z_stroma)){ #this allows to subset the list based on quantile range

z_stroma1[n,2] <- mean(z_stroma[,n])

z_stroma1[n,3] <- quantile(z_stroma[,n],probs=0.25)

z_stroma1[n,4] <- quantile(z_stroma[,n],probs=0.75)

z_stroma1[n,5] <- z_stroma1[n,4]-z_stroma1[n,3]

}

z_stroma2 <- subset(z_stroma1,z_stroma1$V5>0)

stroma_pharm_in_data2 <- as.vector(z_stroma2$gene)

z_stroma3 <- subset(z_stroma1,z_stroma1$V5>variable)

stroma_pharm_in_data3 <- as.vector(z_stroma3$gene)

############## Filtering pharm_matrix_UMInorm down to just lympho regions #################

lympho_barcodes <- list()

for(i in 1:length(sample_names)){

lympho_barcodes[[i]] <- subset(clusters_pathol[[i]],clusters_pathol[[i]][["Pathol"]]=="lympho" |

clusters_pathol[[i]][["Pathol"]]=="Lympho")

}

lympho_pharm_matrix_UMInorm <- list()

for (i in 1:length(sample_names)) {

lympho_pharm_matrix_UMInorm[[i]] <- subset.matrix(pharm_matrix_UMInorm[[i]],pharm_matrix_UMInorm[[i]]@Dimnames[[1]] %in% lympho_barcodes[[i]][["Barcode"]])

}

lympho_pharm_genes <- list() #this creates a list of tables

for (i in 1:length(sample_names)) {

lympho_pharm_genes[[i]] <- as.data.table(lympho_pharm_matrix_UMInorm[[i]])

}

############## Prep for plotting

lympho_pharm_combined <- data.frame() #combines samples

for (i in 1:length(sample_names)) {

skip_to_next <- FALSE

y <- as.data.frame(lympho_pharm_genes[[i]])

tryCatch(y$sample <- sample_names[[i]],error=function(e) { skip_to_next <<- TRUE})

if(skip_to_next) { next }

lympho_pharm_combined <- rbind(lympho_pharm_combined,y)

}

z_lympho <- lympho_pharm_combined[-c(length(lympho_pharm_combined))]

z_lympho1 <- data.frame(colnames(z_lympho))

colnames(z_lympho1) <- "gene"

zz_lympho <- reshape2::melt(lympho_pharm_combined)

for(n in 1:length(z_lympho)){ #this allows to subset the list based on quantile range

z_lympho1[n,2] <- mean(z_lympho[,n])

z_lympho1[n,3] <- quantile(z_lympho[,n],probs=0.25)

z_lympho1[n,4] <- quantile(z_lympho[,n],probs=0.75)

z_lympho1[n,5] <- z_lympho1[n,4]-z_lympho1[n,3]

}

z_lympho2 <- subset(z_lympho1,z_lympho1$V5>0)

lympho_pharm_in_data2 <- as.vector(z_lympho2$gene)

z_lympho3 <- subset(z_lympho1,z_lympho1$V5>variable)

lympho_pharm_in_data3 <- as.vector(z_lympho3$gene)

############## Prep for plotting ###################

# some functions

scale_fill_pharm <- function(...){

ggplot2:::manual_scale(

'fill',

values = setNames(c("goldenrod3","firebrick3","dodgerblue3","green4","darkslategrey"),

c('a1','b1','a2','b2','v1')),

...

)

}

percentile_function <- function(a){

-(quantile(a,probs=0.75,na.rm = T)-quantile(a,probs=0.25,na.rm = T))

}

percentile_function2 <- function(b){

-((quantile(b,probs=0.75,na.rm = T)-quantile(b,probs=0.25,na.rm = T))+(median(b,na.rm=T)/100))

}

############ PLOT Unused ##########################

#box3a <- ggplot(data=zz,mapping=aes(x=variable,y=value))+

# scale_fill_viridis_d()+

# geom_boxplot2()+theme(

# axis.text.x = element_blank(),

# axis.title.y = element_text(size=8),

# axis.title.x = element_blank(),

# axis.ticks.x = element_blank(),

# panel.grid.minor = element_blank(),

# panel.background = element_blank(),

# legend.position = "none")+

# ylab("UMI Normalized\nReads")

#box3a

#ggsave("Unused.tiff",plot = box3a,device = "tiff",dpi=200,units='in',height=4,width = 30)

############ PLOT S3 ##########################

zz_nontumor$group <- "nontumor"

zz_tumor$group <- "tumor"

zz <- rbind(zz_nontumor, zz_tumor)

pharm_in_data2 <- unique(append(nontumor_pharm_in_data2,tumor_pharm_in_data2))

zzz <- zz[zz$variable %in% pharm_in_data2,]

boxs2 <- ggplot(data=zzz,mapping=aes(x=reorder(variable,-value),

y=value,fill=group))+

geom_boxplot2(width.errorbar = 0.3, alpha=0.9)+

theme(axis.text.x = element_text(angle=90,size=8,vjust=.5,hjust = 1),

axis.title.y = element_text(size=8),

axis.text.y = element_blank(),

axis.title.x = element_blank(),

axis.ticks.x = element_blank(),

axis.ticks.y = element_blank(),

panel.grid.minor = element_blank(),

panel.background = element_blank(),

legend.title = element_blank(),

legend.position = c(0.6,0.8),

legend.direction = "horizontal")+

scale_fill_manual(values = c("dodgerblue3","firebrick"))+

#scale_y_continuous(expand = c(0,0),limits = c(0,0.0031))+

ylab("UMI Normalized\nReads")

#boxs2

############ PLOT 3 ##########################

zz_tumor$group <- "tumor"

zz_normal$group <- "normal"

zz_lympho$group <- "lympho"

zz_stroma$group <- "stroma"

zz <- rbind(zz_normal, zz_tumor)

zz <- rbind(zz,zz_lympho)

zz <- rbind(zz,zz_stroma)

pharm_in_data3 <- unique(append(normal_pharm_in_data3,tumor_pharm_in_data3))

pharm_in_data3 <- unique(append(pharm_in_data3,lympho_pharm_in_data3))

pharm_in_data3 <- unique(append(pharm_in_data3,stroma_pharm_in_data3))

zzz <- zz[zz$variable %in% pharm_in_data3,]

box3 <- ggplot(data=zzz,mapping=aes(x=reorder(variable,-value),

y=value,fill=group))+

geom_boxplot2(width.errorbar = 0.3, alpha=0.9)+

theme(axis.text.x = element_text(angle=90,size=8,vjust=.5,hjust = 1),

axis.title.y = element_text(size=8),

axis.text.y = element_blank(),

axis.title.x = element_blank(),

axis.ticks.x = element_blank(),

axis.ticks.y = element_blank(),

panel.grid.minor = element_blank(),

panel.background = element_blank(),

legend.title = element_blank(),

legend.position = c(0.6,0.8),

legend.direction = "horizontal")+

scale_fill_manual(values = c("goldenrod3","dodgerblue3","darkgreen","firebrick"))+

scale_y_continuous(expand = c(0,0),limits = c(0,0.0031))+

ylab("UMI Normalized\nReads")

#box3

### Figure 2A ###################

############## filter Full matrix_UMInorm to pharmacogenes ################################

pharmacogenes <- read.csv("/User/Visium/Transporters and Pharmacogenes (Expanded).csv", header=FALSE)

pharmacogenes <- as.vector(pharmacogenes$V2)

genes_in_data <- vector() #this figures out the genes that are in the matrix_UMInorm data

for (i in 1:length(sample_names)) {

genes_in_data <- append(genes_in_data,matrix_UMInorm[[i]]@Dimnames[[2]])

}

genes_in_data <- unique(genes_in_data)

pharm_in_data <- subset(pharmacogenes,

pharmacogenes %in% genes_in_data) #this picks out the pharamcogenes from our list that are in the data

pharm_matrix_UMInorm <- list() #this creates a matrix_UMInorm for the pharmacogenes

for (i in 1:length(sample_names)) {

pharm_matrix_UMInorm[[i]] <- matrix_UMInorm[[i]][, pharm_in_data, with=FALSE]

}

############## Filtering pharm_matrix_UMInorm down to Tumor #################

tumor_pharm_barcodes <- list()

for(i in 1:length(sample_names)){

tumor_pharm_barcodes[[i]] <- subset(clusters_pathol[[i]],clusters_pathol[[i]][["Pathol"]]=="Tumor" |

clusters_pathol[[i]][["Pathol"]]=="tumor" |

clusters_pathol[[i]][["Pathol"]]=="?DCIS" |

clusters_pathol[[i]][["Pathol"]]=="In situ" |

clusters_pathol[[i]][["Pathol"]]=="Cellular tumor" |

clusters_pathol[[i]][["Pathol"]]=="desmoplastic tumor")

}

tumor_pharm_matrix_UMInorm <- list()

for (i in 1:length(sample_names)) {

tumor_pharm_matrix_UMInorm[[i]] <- subset.matrix(pharm_matrix_UMInorm[[i]],

pharm_matrix_UMInorm[[i]]@Dimnames[[1]] %in% tumor_pharm_barcodes[[i]][["Barcode"]])

}

tumor_pharm_genes <- list() #this creates a list of tables

for (i in 1:length(sample_names)) {

tumor_pharm_genes[[i]] <- as.data.table(tumor_pharm_matrix_UMInorm[[i]])

}

tumor_pharm_combined <- data.frame() #combines samples

for (i in 1:length(sample_names)) {

y <- as.data.frame(tumor_pharm_genes[[i]])

y$sample <- sample_names[[i]]

tumor_pharm_combined <- rbind(tumor_pharm_combined,y)

}

z <- tumor_pharm_combined[-c(length(tumor_pharm_combined))]

z1 <- data.frame(colnames(z))

colnames(z1) <- "gene"

for(n in 1:length(z)){ #gets quantile range for each gene-region pair

z1[n,2] <- mean(z[,n])

z1[n,3] <- quantile(z[,n],probs=0.25)

z1[n,4] <- quantile(z[,n],probs=0.75)

z1[n,5] <- z1[n,4]-z1[n,3]

}

z1 <- z1[c(1,5)]

colnames(z1)[2] <- "Tumor"

tumor <- z1

############## Filtering pharm_matrix_UMInorm down to Normal #################

normal_pharm_barcodes <- list()

for(i in 1:length(sample_names)){

normal_pharm_barcodes[[i]] <- subset(clusters_pathol[[i]],clusters_pathol[[i]][["Pathol"]]=="Normal" |

clusters_pathol[[i]][["Pathol"]]=="normal")

}

normal_pharm_matrix_UMInorm <- list()

for (i in 1:length(sample_names)) {

normal_pharm_matrix_UMInorm[[i]] <- subset.matrix(pharm_matrix_UMInorm[[i]],

pharm_matrix_UMInorm[[i]]@Dimnames[[1]] %in% normal_pharm_barcodes[[i]][["Barcode"]])

}

normal_pharm_genes <- list() #this creates a list of tables

for (i in 1:length(sample_names)) {

normal_pharm_genes[[i]] <- as.data.table(normal_pharm_matrix_UMInorm[[i]])

}

normal_pharm_combined <- data.frame() #combines samples

for (i in 1:length(sample_names)) {

y <- as.data.frame(normal_pharm_genes[[i]])

skip_to_next <- FALSE

tryCatch({y$sample <- sample_names[[i]]},error=function(e){ skip_to_next <<- TRUE})

if(skip_to_next) { next }

normal_pharm_combined <- rbind(normal_pharm_combined,y)

}

za <- normal_pharm_combined[-c(length(normal_pharm_combined))]

za1 <- data.frame(colnames(za))

colnames(za1) <- "gene"

for(n in 1:length(za)){ #gets quantile range for each gene-region pair

za1[n,2] <- mean(za[,n])

za1[n,3] <- quantile(za[,n],probs=0.25)

za1[n,4] <- quantile(za[,n],probs=0.75)

za1[n,5] <- za1[n,4]-za1[n,3]

}

za1 <- za1[c(1,5)]

colnames(za1)[2] <- "Normal"

normal <- za1

############## Filtering pharm_matrix_UMInorm down to Lympho #################

lympho_pharm_barcodes <- list()

for(i in 1:length(sample_names)){

lympho_pharm_barcodes[[i]] <- subset(clusters_pathol[[i]],clusters_pathol[[i]][["Pathol"]]=="lympho" |

clusters_pathol[[i]][["Pathol"]]=="Lympho")

}

lympho_pharm_matrix_UMInorm <- list()

for (i in 1:length(sample_names)) {

lympho_pharm_matrix_UMInorm[[i]] <- subset.matrix(pharm_matrix_UMInorm[[i]],

pharm_matrix_UMInorm[[i]]@Dimnames[[1]] %in% lympho_pharm_barcodes[[i]][["Barcode"]])

}

lympho_pharm_genes <- list() #this creates a list of tables

for (i in 1:length(sample_names)) {

lympho_pharm_genes[[i]] <- as.data.table(lympho_pharm_matrix_UMInorm[[i]])

}

lympho_pharm_combined <- data.frame() #combines samples

for (i in 1:length(sample_names)) {

y <- as.data.frame(lympho_pharm_genes[[i]])

skip_to_next <- FALSE

tryCatch({y$sample <- sample_names[[i]]},error=function(e){ skip_to_next <<- TRUE})

if(skip_to_next) { next }

lympho_pharm_combined <- rbind(lympho_pharm_combined,y)

}

zb <- lympho_pharm_combined[-c(length(lympho_pharm_combined))]

zb1 <- data.frame(colnames(zb))

colnames(zb1) <- "gene"

for(n in 1:length(zb)){ #gets quantile range for each gene-region pair

zb1[n,2] <- mean(zb[,n])

zb1[n,3] <- quantile(zb[,n],probs=0.25)

zb1[n,4] <- quantile(zb[,n],probs=0.75)

zb1[n,5] <- zb1[n,4]-zb1[n,3]

}

zb1 <- zb1[c(1,5)]

colnames(zb1)[2] <- "Lympho"

lympho <- zb1

############## Filtering pharm_matrix_UMInorm down to Stroma #################

stroma_pharm_barcodes <- list()

for(i in 1:length(sample_names)){

stroma_pharm_barcodes[[i]] <- subset(clusters_pathol[[i]],clusters_pathol[[i]][["Pathol"]]=="stroma" |

clusters_pathol[[i]][["Pathol"]]=="Stroma")

}

stroma_pharm_matrix_UMInorm <- list()

for (i in 1:length(sample_names)) {

stroma_pharm_matrix_UMInorm[[i]] <- subset.matrix(pharm_matrix_UMInorm[[i]],

pharm_matrix_UMInorm[[i]]@Dimnames[[1]] %in% stroma_pharm_barcodes[[i]][["Barcode"]])

}

stroma_pharm_genes <- list() #this creates a list of tables

for (i in 1:length(sample_names)) {

stroma_pharm_genes[[i]] <- as.data.table(stroma_pharm_matrix_UMInorm[[i]])

}

stroma_pharm_combined <- data.frame() #combines samples

for (i in 1:length(sample_names)) {

y <- as.data.frame(stroma_pharm_genes[[i]])

skip_to_next <- FALSE

tryCatch({y$sample <- sample_names[[i]]},error=function(e){ skip_to_next <<- TRUE})

if(skip_to_next) { next }

stroma_pharm_combined <- rbind(stroma_pharm_combined,y)

}

zc <- stroma_pharm_combined[-c(length(stroma_pharm_combined))]

zc1 <- data.frame(colnames(zc))

colnames(zc1) <- "gene"

for(n in 1:length(zc)){ #gets quantile range for each gene-region pair

zc1[n,2] <- mean(zc[,n])

zc1[n,3] <- quantile(zc[,n],probs=0.25)

zc1[n,4] <- quantile(zc[,n],probs=0.75)

zc1[n,5] <- zc1[n,4]-zc1[n,3]

}

zc1 <- zc1[c(1,5)]

colnames(zc1)[2] <- "Stroma"

stroma <- zc1

############## merge regions #################

quant <- merge(tumor, normal, by="gene")

quant <- merge(quant,stroma,by="gene")

quant <- merge(quant,lympho,by="gene")

quant$add <- rowSums(quant[,2:5])

quant_subset <- subset(quant, quant$add>0)

quant_subset <- quant_subset[c(1:5)]

quant_subset_melt <- reshape2::melt(quant_subset)

############ PLOT 2A ################

heat2a <- ggplot(quant_subset_melt,aes(y=gene, x=variable,fill=value))+

geom_tile(width=1, height=1)+

theme(axis.text.y = element_text(size=8),

axis.title.x = element_blank(),

axis.ticks.y = element_blank(),

axis.title.y = element_blank(),

legend.title = element_text(size=8),

legend.text = element_blank(),

#legend.text = element_text(size=8,angle=90,hjust = 1),

legend.position = "top")+

scale_fill_gradient(low = muted("dodgerblue4"),high = "goldenrod")+

scale_x_discrete(expand = c(0,0))+labs(fill="Quantile Range of\nUMI Normalized Reads")

#heat2a

#ggsave("Figure2A.tiff",plot=heat2a,device='tiff',dpi=400, units = 'mm',height = 250, width = 100)

### Figure 2B ###################

############## filter Full matrix_UMInorm to pharmacogenes ################################

pharmacogenes <- read.csv("/User/Visium/Transporters and Pharmacogenes (Expanded).csv", header=FALSE)

pharmacogenes <- as.vector(pharmacogenes$V2)

genes_in_data <- vector() #this figures out the genes that are in the matrix_UMInorm data

for (i in 1:length(sample_names)) {

genes_in_data <- append(genes_in_data,matrix_UMInorm[[i]]@Dimnames[[2]])

}

genes_in_data <- unique(genes_in_data)

pharm_in_data <- subset(pharmacogenes,

pharmacogenes %in% genes_in_data) #this picks out the pharamcogenes from our list that are in the data

pharm_matrix_UMInorm <- list() #this creates a matrix_UMInorm for the pharmacogenes

for (i in 1:length(sample_names)) {

pharm_matrix_UMInorm[[i]] <- matrix_UMInorm[[i]][, pharm_in_data, with=FALSE]

}

############## Filtering pharm_matrix_UMInorm down to Tumor #################

tumor_pharm_barcodes <- list()

for(i in 1:length(sample_names)){

tumor_pharm_barcodes[[i]] <- subset(clusters_pathol[[i]],clusters_pathol[[i]][["Pathol"]]=="Tumor" |

clusters_pathol[[i]][["Pathol"]]=="tumor" |

clusters_pathol[[i]][["Pathol"]]=="?DCIS" |

clusters_pathol[[i]][["Pathol"]]=="In situ" |

clusters_pathol[[i]][["Pathol"]]=="Cellular tumor" |

clusters_pathol[[i]][["Pathol"]]=="desmoplastic tumor")

}

tumor_pharm_matrix_UMInorm <- list()

for (i in 1:length(sample_names)) {

tumor_pharm_matrix_UMInorm[[i]] <- subset.matrix(pharm_matrix_UMInorm[[i]],

pharm_matrix_UMInorm[[i]]@Dimnames[[1]] %in% tumor_pharm_barcodes[[i]][["Barcode"]])

}

tumor_pharm_genes <- list() #this creates a list of tables

for (i in 1:length(sample_names)) {

tumor_pharm_genes[[i]] <- as.data.table(tumor_pharm_matrix_UMInorm[[i]])

}

############## divide into samples ###############

x <- list()

for(i in 1:length(sample_names)){

z <- as.data.frame(tumor_pharm_genes[[i]])

z1 <- data.frame(colnames(tumor_pharm_genes[[i]]))

colnames(z1) <- c("gene")

for(n in 1:nrow(z1)){ #gets quantile range for each gene-sample pair

z1[n,2] <- mean(z[,n])

z1[n,3] <- quantile(z[,n],probs=0.25)

z1[n,4] <- quantile(z[,n],probs=0.75)

z1[n,5] <- z1[n,4]-z1[n,3]

}

x[[i]] <- z1

}

############## merge with samples in columns #################

y <- x[[1]]["gene"]

for(n in 1:length(sample_names)){

y[,n+1] <- x[[n]]["V5"]

}

colnames(y)[2:(length(sample_names)+1)] <- sample_names

y$add <- rowSums(y[,2:(length(sample_names)+1)],na.rm = TRUE)

y_subset <- subset(y, y$add>0)

y_subset <- y_subset[c(1:(length(sample_names)+1))]

y_subset_melt <- reshape2::melt(y_subset)

############ PLOT 2B ################

heat2b <- ggplot(y_subset_melt,aes(y=gene, x=variable,fill=value))+

geom_tile(width=1, height=1)+

theme(axis.text.y = element_text(size=8),

axis.title.x = element_blank(),

axis.ticks.y = element_blank(),

axis.title.y = element_blank(),

legend.title = element_text(size=8),

#legend.text = element_text(size=8,angle=90,hjust = 1),

legend.text = element_blank(),

legend.position = "top")+

scale_fill_gradient(low = muted("dodgerblue4"),high = "goldenrod")+

scale_x_discrete(expand = c(0,0))+labs(fill="Quantile Range of\nUMI Normalized Reads")

#heat2b

#ggsave("Figure2B.tiff",plot=heat2b,device='tiff',dpi=400, units = 'mm',height = 250, width = 100)

############# Figure 4 ########################

############## filter Full matrix_UMInorm to pharmacogenes ################################

pharmacogenes <- read.csv("/User/Visium/Transporters and Pharmacogenes (Expanded).csv", header=FALSE)

pharmacogenes <- as.vector(pharmacogenes$V2)

genes_in_data <- vector() #this figures out the genes that are in the matrix_UMInorm data

for (i in 1:length(sample_names)) {

genes_in_data <- append(genes_in_data,matrix_UMInorm[[i]]@Dimnames[[2]])

}

genes_in_data <- unique(genes_in_data)

pharm_in_data <- subset(pharmacogenes,

pharmacogenes %in% genes_in_data) #this picks out the pharamcogenes from our list that are in the data

pharm_matrix_UMInorm <- list() #this creates a matrix for the pharmacogenes

for (i in 1:length(sample_names)) {

pharm_matrix_UMInorm[[i]] <- matrix_UMInorm[[i]][, pharm_in_data, with=FALSE]

}

############## Filtering pharm_matrix_UMInorm down to just tumor + DCIS + In situ regions #################

tumor_barcodes <- list()

for(i in 1:length(sample_names)){

tumor_barcodes[[i]] <- subset(clusters_pathol[[i]],clusters_pathol[[i]][["Pathol"]]=="Tumor" |

clusters_pathol[[i]][["Pathol"]]=="tumor" |

clusters_pathol[[i]][["Pathol"]]=="?DCIS" |

clusters_pathol[[i]][["Pathol"]]=="In situ" |

clusters_pathol[[i]][["Pathol"]]=="Cellular tumor" |

clusters_pathol[[i]][["Pathol"]]=="desmoplastic tumor")

}

tumor_pharm_matrix_UMInorm <- list()

for (i in 1:length(sample_names)) {

tumor_pharm_matrix_UMInorm[[i]] <- subset.matrix(pharm_matrix_UMInorm[[i]],pharm_matrix_UMInorm[[i]]@Dimnames[[1]] %in% tumor_barcodes[[i]][["Barcode"]])

}

tumor_pharm_genes <- list() #this creates a list of tables

for (i in 1:length(sample_names)) {

tumor_pharm_genes[[i]] <- as.matrix(tumor_pharm_matrix_UMInorm[[i]])

}

tumor_pharm_combined <- data.frame() #combines samples

for (i in 1:length(sample_names)) {

y <- as.data.frame(tumor_pharm_genes[[i]])

y$sample <- sample_names[[i]]

tumor_pharm_combined <- rbind(tumor_pharm_combined,y)

}

dif <- list()

for (i in 1:length(sample_names)) {

dif[[i]] <- subset(tumor_pharm_combined,tumor_pharm_combined$sample==sample_names[i])

}

############## Filtering pharm_matrix_UMInorm down to just normal, lympho, and stroma regions #################

nontumor_barcodes <- list()

for(i in 1:length(sample_names)){

nontumor_barcodes[[i]] <- subset(clusters_pathol[[i]],clusters_pathol[[i]][["Pathol"]]=="Normal" |

clusters_pathol[[i]][["Pathol"]]=="normal" |

clusters_pathol[[i]][["Pathol"]]=="lympho" |

clusters_pathol[[i]][["Pathol"]]=="Lympho" |

clusters_pathol[[i]][["Pathol"]]=="stroma" |

clusters_pathol[[i]][["Pathol"]]=="Stroma")

}

nontumor_pharm_matrix_UMInorm <- list()

for (i in 1:length(sample_names)) {

nontumor_pharm_matrix_UMInorm[[i]] <- subset.matrix(pharm_matrix_UMInorm[[i]],pharm_matrix_UMInorm[[i]]@Dimnames[[1]] %in% nontumor_barcodes[[i]][["Barcode"]])

}

nontumor_pharm_genes <- list() #this creates a list of tables

for (i in 1:length(sample_names)) {

nontumor_pharm_genes[[i]] <- as.matrix(nontumor_pharm_matrix_UMInorm[[i]])

}

nontumor_pharm_combined <- data.frame() #combines samples

for (i in 1:length(sample_names)) {

skip_to_next <- FALSE

y <- as.data.frame(nontumor_pharm_genes[[i]])

tryCatch(y$sample <- sample_names[[i]],error=function(e) { skip_to_next <<- TRUE})

if(skip_to_next) { next }

nontumor_pharm_combined <- rbind(nontumor_pharm_combined,y)

}

rat_vector <- vector()

for(n in 1:(length(nontumor_pharm_combined)-1)){

rat_vector[n] <- as.numeric(mean(nontumor_pharm_combined[,n]))

}

############## Differential Expression ####################

f_container <- data.frame()

for(i in 1:length(sample_names)){ #test variances to see what type of t-test to use

f1 <- dif[[i]]

for(n in 1:length(pharm_in_data)){

skip_to_next <- FALSE

tryCatch(f <- var.test(f1[,n],nontumor_pharm_combined[,n]),error=function(e){ skip_to_next <<- TRUE})

if(skip_to_next) { next }

f_container[n,i] <- f[["p.value"]]

}

}

colnames(f_container) <- c(sample_names)

f_shape <- reshape2::melt(f_container)

ggplot()+geom_boxplot(data=f_shape,mapping=aes(x=variable,y=value)) #conclude we need to use a welches t-test

### welches t-test

tp_container <- data.frame()

trat_container <- data.frame()

for(i in 1:length(sample_names)){

t1 <- dif[[i]]

for(n in 1:length(pharm_in_data)){

t <- t.test(t1[,n],nontumor_pharm_combined[,n],var.equal = FALSE)

tp_container[n,i] <- t[["p.value"]]

trat_container[n,i] <- t[["estimate"]][["mean of x"]]/t[["estimate"]][["mean of y"]]

}

}

tratlog <- log2(trat_container)

colnames(tp_container) <- c(sample_names)

colnames(tratlog) <- c(sample_names)

row.names(tp_container) <- pharm_in_data

row.names(tratlog) <- pharm_in_data

tratlog_nan <- data_frame()

for(n in 1:length(pharm_in_data)){

if(all(tratlog[pharm_in_data[n],1:length(sample_names)]=="NaN")) { tratlog_nan <- rbind(tratlog_nan,tratlog[c(pharm_in_data[n]),]) }

}

tratlog <- subset(tratlog, !row.names(tratlog) %in% row.names(tratlog_nan))

tp_container_bonferroni <- tp_container*(nrow(tratlog))*(length(sample_names))

tp_container_bonferroni$gene <- row.names(tp_container_bonferroni)

tratlog$gene <- row.names(tratlog)

t_shape <- reshape2::melt(tp_container_bonferroni)

t_shape$name <- paste0(t_shape$gene,t_shape$variable)

ggplot()+geom_boxplot(data=t_shape,mapping=aes(x=variable,y=value))

tratlog_shape <- reshape2::melt(tratlog)

tratlog_shape$name <- paste0(tratlog_shape$gene,tratlog_shape$variable)

ggplot()+geom_boxplot(data=tratlog_shape,mapping=aes(x=variable,y=value))

tlog_final <- merge(tratlog_shape,t_shape,by="name")

tlog_final <- tlog_final[-c(5:6)]

tlog_final$value.x <- as.numeric(gsub("-Inf",min(tlog_final$value.x[is.finite(tlog_final$value.x)])-2, tlog_final$value.x))

tlog_final$value.x <- as.numeric(gsub("Inf",max(tlog_final$value.x[is.finite(tlog_final$value.x)])+2, tlog_final$value.x))

tlog_final$value.x <- as.numeric(gsub("NaN","0", tlog_final$value.x))

colnames(tlog_final)[2:5] <- c("Gene","Sample","Log2_Fold_Change","p_value")

tlog_abc <- subset(tlog_final, grepl("ABC",tlog_final$Gene))

tlog_top_genes <- subset(tlog_final, tlog_final$p_value<0.0000000000000000000000000005)

tlog_tops <- subset(tlog_final,tlog_final$Gene %in% tlog_top_genes$Gene)

tlog_ro <- subset(tlog_final,

tlog_final$Gene=="ALDH3B1" |

tlog_final$Gene=="ARNT" |

tlog_final$Gene=="ATP7A" |

tlog_final$Gene=="CAT" |

tlog_final$Gene=="CBR1" |

tlog_final$Gene=="DHRS2" |

tlog_final$Gene=="DHRS4" |

tlog_final$Gene=="FMO1" |

tlog_final$Gene=="FMO2" |

tlog_final$Gene=="FMO3" |

tlog_final$Gene=="FMO4" |

tlog_final$Gene=="FMO5" |

tlog_final$Gene=="FMO6P" |

tlog_final$Gene=="GPX1" |

tlog_final$Gene=="GPX2" |

tlog_final$Gene=="GPX3" |

tlog_final$Gene=="GPX4" |

tlog_final$Gene=="GPX5" |

tlog_final$Gene=="GPX6" |

tlog_final$Gene=="GPX7" |

tlog_final$Gene=="GSR" |

tlog_final$Gene=="GSS" |

tlog_final$Gene=="GSTP1" |

tlog_final$Gene=="MGST1" |

tlog_final$Gene=="MPO" |

tlog_final$Gene=="NOS1" |

tlog_final$Gene=="NOS3" |

tlog_final$Gene=="PON2" |

tlog_final$Gene=="PON3" |

tlog_final$Gene=="POR" |

tlog_final$Gene=="PPARA" |

tlog_final$Gene=="SOD1" |

tlog_final$Gene=="SOD2" |

tlog_final$Gene=="SOD3"

)

tlog_nonabc <- subset(tlog_final,

# tlog_final$Gene=="ATP7A" |

# tlog_final$Gene=="ATP7B" |

# tlog_final$Gene=="CFTR" |

# tlog_final$Gene=="SERPINA7" |

# tlog_final$Gene=="TAP1" |

# tlog_final$Gene=="TAP2" |

# tlog_final$Gene=="KCNJ11" |

grepl("SLC",tlog_final$Gene)

)

############## Plots ####################

s4a <- ggplot()+geom_hline(yintercept = 0,linetype="dashed",color="black",size=0.3,alpha=0.5)+

geom_point(data=tlog_final,

mapping=aes(y=Log2_Fold_Change,x=reorder(name,Log2_Fold_Change),

color=p_value,

shape=Sample),alpha=0.8,size=0.8)+

scale_color_gradientn(colours=c("firebrick","goldenrod4"),

limits=c(0,0.05),

breaks=c(0,0.025,0.05),

na.value="grey")+

theme(axis.text.x = element_blank(),

panel.background = element_rect(fill = "white"),

panel.grid = element_blank(),

axis.line = element_line(size=0.5),

axis.ticks.y = element_blank(),

axis.ticks.x = element_blank(),

legend.title = element_text(size=8),

legend.text = element_text(size=8),

axis.title = element_text(size=8),

axis.title.x = element_text(vjust = 25))+

scale_y_continuous(breaks = seq(-9,7,by=2),limits = c(-12,12))+

scale_x_discrete(expand=c(0.03,0))+

xlab("Gene-Sample Pair")+

ylab("Log2-Fold Change")+

geom_hline(yintercept = -10,color="black",size=0.5)+

geom_hline(yintercept = 9.5,color="black",size=0.5)

#s4a

#ggsave("Figure4a.tiff",plot=s4a,device='tiff',dpi=400, units = 'mm',height = 186, width = 186)

s4b <- ggplot()+geom_hline(yintercept = 0,linetype="dashed",color="black",size=0.3,alpha=0.5)+

geom_point(data=tlog_final,

mapping=aes(y=Log2_Fold_Change,x=reorder(Gene,Log2_Fold_Change,FUN = mean),

color=p_value,

shape=Sample),alpha=0.8,size=0.8)+

scale_color_gradientn(colours=c("firebrick","goldenrod"),

limits=c(0,0.05),

breaks=c(0,0.025,0.05),

na.value="grey")+

theme(axis.text.x = element_blank(),

panel.background = element_rect(fill = "white"),

panel.grid = element_blank(),

axis.line = element_line(size=0.5),

axis.ticks.y = element_blank(),

axis.ticks.x = element_blank(),

legend.title = element_text(size=8),

legend.text = element_text(size=8),

axis.title = element_text(size=8),

axis.title.x = element_text(vjust = 25))+

scale_y_continuous(breaks = seq(-9,7,by=2),limits = c(-12,12))+

scale_x_discrete(expand=c(0.03,0))+

xlab("Gene")+ylab("Log2-Fold Change")+

geom_hline(yintercept = -10,color="black",size=0.5)+

geom_hline(yintercept = 9.5,color="black",size=0.5)

#s4b

#ggsave("Figure4b.tiff",plot=s4b,device='tiff',dpi=400, units = 'mm',height = 186, width = 186)

s4d <- ggplot()+geom_hline(yintercept = 0,linetype="dashed",color="black",size=0.3,alpha=0.5)+

geom_point(data=tlog_ro,

mapping=aes(y=Log2_Fold_Change,x=reorder(Gene,Log2_Fold_Change,FUN = mean),

color=p_value,

shape=Sample),

alpha=0.8,

size=2)+

scale_color_gradientn(colours=c("firebrick","goldenrod"),

limits=c(0,0.05),

breaks=c(0,0.025,0.05),

na.value="grey")+

theme(axis.text.x = element_text(size=8,angle=90,vjust = 0.5),

panel.background = element_rect(fill = "white"),

axis.text.y = element_text(size=8),

axis.title = element_text(size=8),

panel.grid = element_blank(),

axis.line = element_line(size=0.5),

axis.ticks.y = element_blank(),

axis.ticks.x = element_blank(),

legend.title = element_text(size=8),

legend.text = element_text(size=8),

axis.title.x = element_blank())+

scale_y_continuous(breaks = seq(-9,7,by=2),limits = c(-12,12))+

scale_x_discrete(expand=c(0.05,0))+

ylab("Log2-Fold Change")+

geom_hline(yintercept = -10,color="black",size=0.5)+

geom_hline(yintercept = 9.5,color="black",size=0.5)

#s4d

#ggsave("Figure4d.tiff",plot=s4d,device='tiff',dpi=400, units = 'mm',height = 186, width = 186)

s4c <- ggplot()+geom_hline(yintercept = 0,linetype="dashed",color="black",size=0.3,alpha=0.5)+

geom_point(data=tlog_tops,

mapping=aes(y=Log2_Fold_Change,x=reorder(Gene,Log2_Fold_Change,FUN = mean),

color=p_value,

shape=Sample),alpha=0.8,size=1.5)+

scale_color_gradientn(colours=c("firebrick","goldenrod"),

limits=c(0,0.05),

breaks=c(0,0.025,0.05),

na.value="grey")+

theme(axis.text.x = element_text(size=8,angle=90,vjust=0.5),

panel.background = element_rect(fill = "white"),

panel.grid = element_blank(),

axis.line = element_line(size=0.5),

axis.ticks.y = element_blank(),

axis.ticks.x = element_blank(),

legend.title = element_text(size=8),

legend.text = element_text(size=8),

axis.title = element_text(size=8),

axis.title.x = element_blank())+

scale_y_continuous(breaks = seq(-9,7,by=2),limits = c(-12,12))+

scale_x_discrete(expand=c(0.03,0))+

ylab("Log2-Fold Change")+

geom_hline(yintercept = -10,color="black",size=0.5)+

geom_hline(yintercept = 9.5,color="black",size=0.5)

#s4c

#ggsave("Figure4c.tiff",plot=s4c,device='tiff',dpi=400, units = 'mm',height = 186, width = 186)

s4e <- ggplot()+geom_hline(yintercept = 0,linetype="dashed",color="black",size=0.3,alpha=0.5)+

geom_point(data=tlog_abc,

mapping=aes(y=Log2_Fold_Change,x=reorder(Gene,Log2_Fold_Change,FUN = mean),

color=p_value,

shape=Sample),

alpha=0.8,

size=2)+

scale_color_gradientn(colours=c("firebrick","goldenrod"),

limits=c(0,0.05),

breaks=c(0,0.025,0.05),

na.value="grey")+

theme(axis.text.x = element_text(size=8,angle=90,vjust = 0.5),

panel.background = element_rect(fill = "white"),

axis.text.y = element_text(size=8),

axis.title = element_text(size=8),

panel.grid = element_blank(),

axis.line = element_line(size=0.5),

axis.ticks.y = element_blank(),

axis.ticks.x = element_blank(),

legend.title = element_text(size=8),

legend.text = element_text(size=8),

axis.title.x = element_blank())+

scale_y_continuous(breaks = seq(-9,7,by=2),limits = c(-12,12))+

scale_x_discrete(expand=c(0.03,0))+

ylab("Log2-Fold Change")+

geom_hline(yintercept = -10,color="black",size=0.5)+

geom_hline(yintercept = 9.5,color="black",size=0.5)

#s4e

#ggsave("Figure4e.tiff",plot=s4e,device='tiff',dpi=400, units = 'mm',height = 186, width = 186)

s4f <- ggplot()+geom_hline(yintercept = 0,linetype="dashed",color="black",size=0.3,alpha=0.5)+

geom_point(data=tlog_nonabc,

mapping=aes(y=Log2_Fold_Change,x=reorder(Gene,Log2_Fold_Change,FUN = mean),

color=p_value,

shape=Sample),

alpha=0.8,

size=2)+

scale_color_gradientn(colours=c("firebrick","goldenrod"),

limits=c(0,0.05),

breaks=c(0,0.025,0.05),

na.value="grey")+

theme(axis.text.x = element_text(size=8,angle=90,vjust = 0.5),

panel.background = element_rect(fill = "white"),

axis.text.y = element_text(size=8),

axis.title = element_text(size=8),

panel.grid = element_blank(),

axis.line = element_line(size=0.5),

axis.ticks.y = element_blank(),

axis.ticks.x = element_blank(),

legend.title = element_text(size=8),

legend.text = element_text(size=8),

axis.title.x = element_blank())+

scale_y_continuous(breaks = seq(-9,7,by=2),limits = c(-12,12))+

scale_x_discrete(expand=c(0.03,0))+

ylab("Log2-Fold Change")+

geom_hline(yintercept = -10,color="black",size=0.5)+

geom_hline(yintercept = 9.5,color="black",size=0.5)

#s4f

#ggsave("Figure4f.tiff",plot=s4f,device='tiff',dpi=400, units = 'mm',height = 186, width = 186)

# ############# Figure 6 ########################

#

# ############## filter Full matrix to pharmacogenes ################################

# pharmacogenes <- read.csv("/User/Visium/Transporters and Pharmacogenes (Expanded).csv", header=FALSE)

# pharmacogenes <- as.vector(pharmacogenes$V2)

#

# genes_in_data <- vector() #this figures out the genes that are in the matrix data

# for (i in 1:length(sample_names)) {

# genes_in_data <- append(genes_in_data,matrix[[i]]@Dimnames[[2]])

# }

# genes_in_data <- unique(genes_in_data)

# pharm_in_data <- subset(pharmacogenes,

# pharmacogenes %in% genes_in_data) #this picks out the pharamcogenes from our list that are in the data

#

# pharm_matrix <- list() #this creates a matrix for the pharmacogenes

# for (i in 1:length(sample_names)) {

# pharm_matrix[[i]] <- matrix[[i]][, pharm_in_data, with=FALSE]

# }

# ############## Filtering pharm_matrix down to just tumor + DCIS + In situ regions #################

# tumor_barcodes <- list()

# for(i in 1:length(sample_names)){

# tumor_barcodes[[i]] <- subset(clusters_pathol[[i]],clusters_pathol[[i]][["Pathol"]]=="Tumor" |

# clusters_pathol[[i]][["Pathol"]]=="tumor" |

# clusters_pathol[[i]][["Pathol"]]=="?DCIS" |

# clusters_pathol[[i]][["Pathol"]]=="In situ" |

# clusters_pathol[[i]][["Pathol"]]=="Cellular tumor" |

# clusters_pathol[[i]][["Pathol"]]=="desmoplastic tumor")

# }

#

# tumor_pharm_matrix <- list()

# for (i in 1:length(sample_names)) {

# tumor_pharm_matrix[[i]] <- subset.matrix(pharm_matrix[[i]],pharm_matrix[[i]]@Dimnames[[1]] %in% tumor_barcodes[[i]][["Barcode"]])

# }

#

# tumor_pharm_genes <- list() #this creates a list of tables

# for (i in 1:length(sample_names)) {

# tumor_pharm_genes[[i]] <- as.data.table(tumor_pharm_matrix[[i]])

# }

#

# tumor_pharm_combined <- data.frame() #combines samples

# for (i in 1:length(sample_names)) {

# y <- as.data.frame(tumor_pharm_genes[[i]])

# y$sample <- sample_names[[i]]

# tumor_pharm_combined <- rbind(tumor_pharm_combined,y)

# }

#

# rat_vector <- vector()

# for(n in 1:(length(nontumor_pharm_combined)-1)){

# rat_vector[n] <- as.numeric(mean(nontumor_pharm_combined[,n]))

# }

#

# dif <- list()

# for (i in 1:length(sample_names)) {

# dif[[i]] <- subset(tumor_pharm_combined,tumor_pharm_combined$sample==sample_names[i])

# }

#

# ############## Filtering pharm_matrix down to just normal, lympho, and stroma regions #################

# nontumor_barcodes <- list()

# for(i in 1:length(sample_names)){

# nontumor_barcodes[[i]] <- subset(clusters_pathol[[i]],clusters_pathol[[i]][["Pathol"]]=="Normal" |

# clusters_pathol[[i]][["Pathol"]]=="normal" |

# clusters_pathol[[i]][["Pathol"]]=="lympho" |

# clusters_pathol[[i]][["Pathol"]]=="Lympho" |

# clusters_pathol[[i]][["Pathol"]]=="stroma" |

# clusters_pathol[[i]][["Pathol"]]=="Stroma")

#

# }

#

# nontumor_pharm_matrix <- list()

# for (i in 1:length(sample_names)) {

# nontumor_pharm_matrix[[i]] <- subset.matrix(pharm_matrix[[i]],pharm_matrix[[i]]@Dimnames[[1]] %in% nontumor_barcodes[[i]][["Barcode"]])

# }

#

# nontumor_pharm_genes <- list() #this creates a list of tables

# for (i in 1:length(sample_names)) {

# nontumor_pharm_genes[[i]] <- as.data.table(nontumor_pharm_matrix[[i]])

# }

#

# nontumor_pharm_combined <- data.frame() #combines samples

# for (i in 1:length(sample_names)) {

# skip_to_next <- FALSE

# y <- as.data.frame(nontumor_pharm_genes[[i]])

# tryCatch(y$sample <- sample_names[[i]],error=function(e) { skip_to_next <<- TRUE})

# if(skip_to_next) { next }

# nontumor_pharm_combined <- rbind(nontumor_pharm_combined,y)

# }

#

#

# ############## Differential Expression ####################

# f_container <- data.frame()

# for(i in 1:length(sample_names)){ #test variances to see what type of t-test to use

# f1 <- dif[[i]]

# for(n in 1:length(pharm_in_data)){

# skip_to_next <- FALSE

# tryCatch(f <- var.test(f1[,n],nontumor_pharm_combined[,n]),error=function(e){ skip_to_next <<- TRUE})

# if(skip_to_next) { next }

# f_container[n,i] <- f[["p.value"]]

# }

# }

# colnames(f_container) <- c(sample_names)

#

# f_shape <- reshape2::melt(f_container)

# ggplot()+geom_boxplot(data=f_shape,mapping=aes(x=variable,y=value)) #conclude we need to use a welches t-test

#

# ### welches t-test

# tp_container <- data.frame()

# trat_container <- data.frame()

# for(i in 1:length(sample_names)){

# t1 <- dif[[i]]

# for(n in 1:length(pharm_in_data)){

# t <- t.test(t1[,n],nontumor_pharm_combined[,n],var.equal = FALSE)

# tp_container[n,i] <- t[["p.value"]]

# trat_container[n,i] <- t[["estimate"]][["mean of x"]]/t[["estimate"]][["mean of y"]]

# }

# }

#

# tratlog <- log2(trat_container)

# colnames(tp_container) <- c(sample_names)

# colnames(tratlog) <- c(sample_names)

# row.names(tp_container) <- pharm_in_data

# row.names(tratlog) <- pharm_in_data

# tp_container_bonferroni <- tp_container*286*(length(sample_names))

# tp_container_bonferroni$gene <- row.names(tp_container_bonferroni)

# tratlog$gene <- row.names(tratlog)

# t_shape <- reshape2::melt(tp_container_bonferroni)

# t_shape$name <- paste0(t_shape$gene,t_shape$variable)

# ggplot()+geom_boxplot(data=t_shape,mapping=aes(x=variable,y=value))

#

# tratlog_shape <- reshape2::melt(tratlog)

# tratlog_shape$name <- paste0(tratlog_shape$gene,tratlog_shape$variable)

# ggplot()+geom_boxplot(data=tratlog_shape,mapping=aes(x=variable,y=value))

#

# tlog_final <- merge(tratlog_shape,t_shape,by="name")

# tlog_final <- tlog_final[-c(5:6)]

# tlog_final$value.x <- as.numeric(gsub("-Inf",min(tlog_final$value.x[is.finite(tlog_final$value.x)])-2, tlog_final$value.x))

# tlog_final$value.x <- as.numeric(gsub("Inf",max(tlog_final$value.x[is.finite(tlog_final$value.x)])+2, tlog_final$value.x))

# colnames(tlog_final)[2:5] <- c("Gene","Sample","Log2_Fold_Change","p_value")

#

# tlog_abc <- subset(tlog_final, grepl("ABC",tlog_final$Gene))

# tlog_top_genes <- subset(tlog_final,

# tlog_final$p_value<1e-150)

# tlog_tops <- subset(tlog_final,tlog_final$Gene %in% tlog_top_genes$Gene)

#

# ############## Plots ####################

# s6a <- ggplot()+geom_point(data=tlog_final,

# mapping=aes(y=Log2_Fold_Change,x=reorder(name,Log2_Fold_Change),

# color=p_value,

# shape=Sample),alpha=0.8,size=0.8)+

# scale_color_gradientn(colours=c("firebrick","goldenrod4"),

# limits=c(0,0.05),

# breaks=c(0,0.025,0.05),

# na.value="grey")+

# theme(axis.text.x = element_blank(),

# #panel.background = element_rect(fill = "red"),

# axis.line = element_line(size=0.5),

# axis.ticks.y = element_blank(),

# axis.ticks.x = element_blank(),

# legend.title = element_text(size=8),

# legend.text = element_text(size=8),

# axis.title = element_text(size=8))+

# scale_y_continuous(breaks = seq(-7,7,by=2))+

# xlab("Gene-Sample Pair")+ylab("Log2-Fold Change")+

# geom_hline(yintercept = -7.5,color="black",size=0.5)+

# geom_hline(yintercept = 7,color="black",size=0.5)

# #s6a

# ggsave("Figure6a.tiff",plot=s6a,device='tiff',dpi=400, units = 'mm',height = 186, width = 186)

#

# s6b <- ggplot()+geom_point(data=tlog_final,

# mapping=aes(y=Log2_Fold_Change,x=reorder(Gene,Log2_Fold_Change,FUN = mean),

# color=p_value,

# shape=Sample),alpha=0.8,size=0.8)+

# scale_color_gradientn(colours=c("firebrick","goldenrod"),

# limits=c(0,0.05),

# breaks=c(0,0.025,0.05),

# na.value="grey")+

# theme(axis.text.x = element_blank(),

# #panel.background = element_rect(fill = "red"),

# panel.grid = element_blank(),

# axis.line = element_line(size=0.5),

# axis.ticks.y = element_blank(),

# axis.ticks.x = element_blank(),

# legend.title = element_text(size=8),

# legend.text = element_text(size=8),

# axis.title = element_text(size=8))+

# scale_y_continuous(breaks = seq(-9,7,by=2))+

# xlab("Gene")+ylab("Log2-Fold Change")+

# geom_hline(yintercept = -7.5,color="white",size=2)+

# geom_hline(yintercept = 7,color="white",size=2)

# #s6b

# ggsave("Figure6b.tiff",plot=s6b,device='tiff',dpi=400, units = 'mm',height = 186, width = 186)

#

# s6c <- ggplot()+geom_hline(yintercept = 0,linetype="dashed",color="black",size=0.3,alpha=0.5)+

# geom_point(data=tlog_abc,

# mapping=aes(y=Log2_Fold_Change,x=reorder(Gene,Log2_Fold_Change,FUN = mean),

# color=p_value,

# shape=Sample),

# alpha=0.8,

# size=2)+

# scale_color_gradientn(colours=c("firebrick","goldenrod"),

# limits=c(0,0.05),

# breaks=c(0,0.025,0.05),

# na.value="grey")+

# theme(axis.text.x = element_text(size=8,angle=90,vjust = 0.5),

# #panel.background = element_rect(fill = "red"),

# axis.text.y = element_text(size=8),

# axis.title = element_text(size=8),

# panel.grid = element_blank(),

# axis.line = element_line(size=0.5),

# axis.ticks.y = element_blank(),

# axis.ticks.x = element_blank(),

# legend.title = element_text(size=8),

# legend.text = element_text(size=8),

# axis.title.x = element_blank())+

# scale_y_continuous(breaks = seq(-9,7,by=2))+

# ylab("Log2-Fold Change")+

# geom_hline(yintercept = -7.5,color="white",size=2)+

# geom_hline(yintercept = 7,color="white",size=2)

#

# #s6c

# ggsave("Figure6c.tiff",plot=s6c,device='tiff',dpi=400, units = 'mm',height = 186, width = 186)

#

# s6d <- ggplot()+geom_hline(yintercept = 0,linetype="dashed",color="black",size=0.3,alpha=0.5)+

# geom_point(data=tlog_tops,

# mapping=aes(y=Log2_Fold_Change,x=reorder(Gene,Log2_Fold_Change,FUN = mean),

# color=p_value,

# shape=Sample),alpha=0.8,size=1.5)+

# scale_color_gradientn(colours=c("firebrick","goldenrod"),

# limits=c(0,0.05),

# breaks=c(0,0.025,0.05),

# na.value="grey")+

# theme(axis.text.x = element_text(size=8,angle=90,vjust=0.5),

# #panel.background = element_rect(fill = "red"),

# panel.grid = element_blank(),

# axis.line = element_line(size=0.5),

# axis.ticks.y = element_blank(),

# axis.ticks.x = element_blank(),

# legend.title = element_text(size=8),

# legend.text = element_text(size=8),

# axis.title = element_text(size=8),

# axis.title.x = element_blank())+

# scale_y_continuous(breaks = seq(-9,7,by=2))+

# ylab("Log2-Fold Change")+

# geom_hline(yintercept = -7.5,color="white",size=2)+

# geom_hline(yintercept = 7,color="white",size=2)

# #s6d

# ggsave("Figure6d.tiff",plot=s6d,device='tiff',dpi=400, units = 'mm',height = 186, width = 186)

############## Gene Counts ##########################################

#this code gets total spots count used in manuscript

for (n in 1:length(sample_names)) {

x <- nrow(clusters_pathol[[n]])

x1[n,1] <- x

}

total_spots <- sum(x1$V1)

x1 <- data.frame()

for(n in 1:length(sample_names)){

x1[n,1] <- nrow(tumor_pharm_barcodes[[n]])

}

total_tumor_spots <- sum(x1$V1)

x1 <- data.frame()

for(n in 1:length(sample_names)){

x1[n,1] <- nrow(stroma_pharm_barcodes[[n]])

}

total_stroma_spots <- sum(x1$V1)

x1 <- data.frame()

for(n in 1:length(sample_names)){

x1[n,1] <- nrow(lympho_pharm_barcodes[[n]])

}

total_lympho_spots <- sum(x1$V1)

x1 <- data.frame()

for(n in 1:length(sample_names)){

x1[n,1] <- nrow(normal_pharm_barcodes[[n]])

}

total_normal_spots <- sum(x1$V1)

pharm_gene_counts <- as.data.frame(colnames(pharm_matrix[[1]]))

colnames(pharm_gene_counts) <- "x"

for (n in 1:length(sample_names)) {

x <- colnames(pharm_matrix[[n]])

y <- colSums(pharm_matrix[[n]])

z <- data.frame(x,y)

pharm_gene_counts <- merge(pharm_gene_counts,z,by="x")

}

pharm_gene_counts <- pharm_gene_counts[!duplicated(pharm_gene_counts$x),]

row.names(pharm_gene_counts) <- pharm_gene_counts$x

pharm_gene_counts <- pharm_gene_counts[-c(1)]

colnames(pharm_gene_counts) <- sample_names

pharm_gene_counts$total <- rowSums(pharm_gene_counts)

pharm_gene_counts <- pharm_gene_counts[order(pharm_gene_counts$total,decreasing = TRUE),]

#write.csv(pharm_gene_counts,"pharm_gene_counts.csv")

x <- subset(pharm_gene_counts,pharm_gene_counts$total>0) #number used in manuscript

x <- data.frame()

for(n in 1:nrow(pharm_gene_counts)){

if(all(pharm_gene_counts[n,]>0)) { x[n,1] <- 1 } #number used in manuscript (pharmacogenes expressed in all tissues)

}

#this gets more descriptive data

gene_counts <- as.data.frame(colnames(matrix[[1]]))

colnames(gene_counts) <- "x"

for (n in 1:length(sample_names)) {

x <- colnames(matrix[[n]])

y <- colSums(matrix[[n]])

z <- data.frame(x,y)

gene_counts <- merge(gene_counts,z,by="x")

}

gene_counts <- gene_counts[!duplicated(gene_counts$x),] # this is total counts per gene per sample

row.names(gene_counts) <- gene_counts$x

gene_counts <- gene_counts[-c(1)]

colnames(gene_counts) <- sample_names

gene_counts$total <- rowSums(gene_counts) # this adds up the total for each gene across samples as a way to remove zeros

gene_counts <- gene_counts[order(gene_counts$total,decreasing = TRUE),]

#write.csv(gene_counts,"gene_counts.csv")

x <- subset(gene_counts, gene_counts$total!=0)

gene_counts$gene <- row.names(gene_counts)

gene_counts <- gene_counts[-c(7)]

gene_counts_perspot <- gene_counts

for(n in 1:length(sample_names)){

gene_counts_perspot[,n] <- gene_counts[,n]/nrow(matrix[[n]]) #this normalizes to the number of total spots

}

for(n in 1:nrow(gene_counts_perspot)){

gene_counts_perspot[n,8] <- gene_counts_perspot[n,7] %in% pharm_in_data

}

colnames(gene_counts_perspot)[8] <- "pharm"

gene_counts_perspot_melt <- reshape2::melt(gene_counts_perspot)

gene_counts_perspot_melt_log <- gene_counts_perspot_melt

gene_counts_perspot_melt_log$value <- log(gene_counts_perspot_melt_log$value)

gene_counts_perspot_melt_log_pharm <- subset(gene_counts_perspot_melt_log, gene_counts_perspot_melt_log$pharm==TRUE)

for(n in 1:nrow(gene_counts_perspot_melt_log_pharm)){

if(gene_counts_perspot_melt_log_pharm[n,1] %in% pharm_in_data) { gene_counts_perspot_melt_log_pharm[n,5] <- "Other Pharmacogene" }

if(grepl("CYP",gene_counts_perspot_melt_log_pharm[n,1])) { gene_counts_perspot_melt_log_pharm[n,5] <- "CYP450" }

if(gene_counts_perspot_melt_log_pharm[n,1] %in% tlog_abc$Gene) { gene_counts_perspot_melt_log_pharm[n,5] <- "ABC Transporter" }

if(gene_counts_perspot_melt_log_pharm[n,1] %in% tlog_nonabc$Gene) { gene_counts_perspot_melt_log_pharm[n,5] <- "Non-ABC Transporter" }

if(gene_counts_perspot_melt_log_pharm[n,1] %in% tlog_ro$Gene) { gene_counts_perspot_melt_log_pharm[n,5] <- "RO Handling Gene" }

}

############## Plot Gene Counts ############################

gene_counts_perspot_melt_log_pharm$V5[gene_counts_perspot_melt_log_pharm$V5=="CYP450"] <- "CYP"

supp_counta <- ggplot()+geom_point(data=gene_counts_perspot_melt_log,

mapping=aes(y=value,x=reorder(gene,-value),

color="All Genes",

shape=variable),alpha=0.5,size=0.8)+

scale_color_manual(values = c("grey","firebrick4"))+

# limits=c(0,0.05),

# breaks=c(0,0.025,0.05),

# na.value="grey")+

theme(axis.text.x = element_blank(),

axis.text.y = element_blank(),

panel.background = element_rect(fill = "white"),

panel.grid = element_blank(),

axis.line = element_line(size=0.5),

legend.title = element_blank(),

axis.ticks.y = element_blank(),

axis.ticks.x = element_blank(),

legend.text = element_text(size=8),

axis.title = element_text(size=8)

)+

geom_point(data=gene_counts_perspot_melt_log_pharm,

mapping=aes(y=value,x=reorder(gene,-value),

color="Pharmacogenes",

shape=variable),alpha=0.5,size=0.8)+

xlab("Gene")+ylab("Relative Expression")+

guides(shape=guide_none())

supp_countb <- ggplot()+geom_point(data=gene_counts_perspot_melt_log_pharm,

mapping=aes(y=value,x=reorder(gene,-value),

color=V5,

shape=variable),alpha=0.8,size=1)+

scale_color_manual(values = c("firebrick3","dodgerblue3","goldenrod3","grey","darkgreen"))+

# limits=c(0,0.05),

# breaks=c(0,0.025,0.05),

# na.value="grey")+

theme(axis.text.x = element_text(size = 4,angle = 90),

axis.text.y = element_blank(),

panel.background = element_rect(fill = "white"),

panel.grid = element_blank(),

axis.line = element_line(size=0.5),

axis.ticks.y = element_blank(),

axis.ticks.x = element_blank(),

legend.title = element_blank(),

legend.text = element_text(size=8),

axis.title = element_text(size=8),

legend.position ="top",

legend.box ="vertical",

legend.direction = "horizontal")+

guides(shape=guide_legend(nrow = 1))+

xlab("Gene")+ylab("Relative Expression")

############ Arrange Figures for Pub #######################

#ggsave("Figure1.tiff",plot = box1b,device = "tiff",dpi=400,units='in',height=4,width = 10)

ggarrange(heat2d,heat2b,heat2c,heat2a,ncol = 4,labels = "AUTO",legend = "top")

#ggsave("Figure2.tiff",plot=last_plot(),device='tiff',dpi=400, units = 'mm',height = 350, width = 350)

#ggsave("Figure3.tiff",plot = box3,device = "tiff",dpi=400,units='in',height=4,width = 10)

ggarrange(s4a,s4b,s4c,s4d,s4e,s4f,ncol = 3,nrow=2,labels = "AUTO",legend = "top",common.legend = TRUE,widths = c(1,1,1),align = "h")

#ggsave("Figure4.tiff",plot=last_plot(),device='tiff',dpi=400, units = 'mm',height = 186, width = 350)

ggarrange(supp_counta,supp_countb,nrow=2,labels = "AUTO",legend = "top",common.legend = FALSE)

ggsave("FigureS1.tiff",plot=last_plot(),device='tiff',dpi=400, units = 'mm',height = 186, width = 350)

#ggsave("FigureS3.tiff",plot = boxs2,device = "tiff",dpi=400,units='in',height=4,width = 10)
