# Supplementary material for: Quantification of spatial pharmacogene expression heterogeneity in breast tumors
Source: Cancer Rep (Hoboken). 2022 Jul 30;6(1):e1686. doi: 10.1002/cnr2.1686 (PMC9875649; doi:10.1002/cnr2.1686)
Supplement: Supplementary file 2 — Figure S1: Comparison of expression levels of pharmacogenes. Expression is plotted on the y‐axis as the log10 of mRNA reads per sample normalized to the total number of spots for that sample for each gene on the x‐axis. Order is determined by average of the relative expression in each group. (A) All genes in gray versus pharmacogenes in red. (B) Pharmacogenes divided by category. Figure S2: Tissue images showing the barcoded dots where combined unique‐UMI reads of the ABC transporters are greater than 0. Color intensity represents number of reads, and circles with black outlines denote the regions that were pathologist annotated as tumor. Figure S3: Boxplots of UMI‐normalized unique‐UMI reads for subsets of pharmacogenes with the largest interquartile ranges. Groups divided into tumor (tumor + DCIS + cellular tumor + desmoplastic tumor) and non‐tumor (normal + lymphocytes + stroma), showing the gene subset based on interquartile ranges greater than 0. Y‐axis scale is arbitrary, as this figure is intended to show relative differences. [file CNR2-6-e1686-s001.pdf]

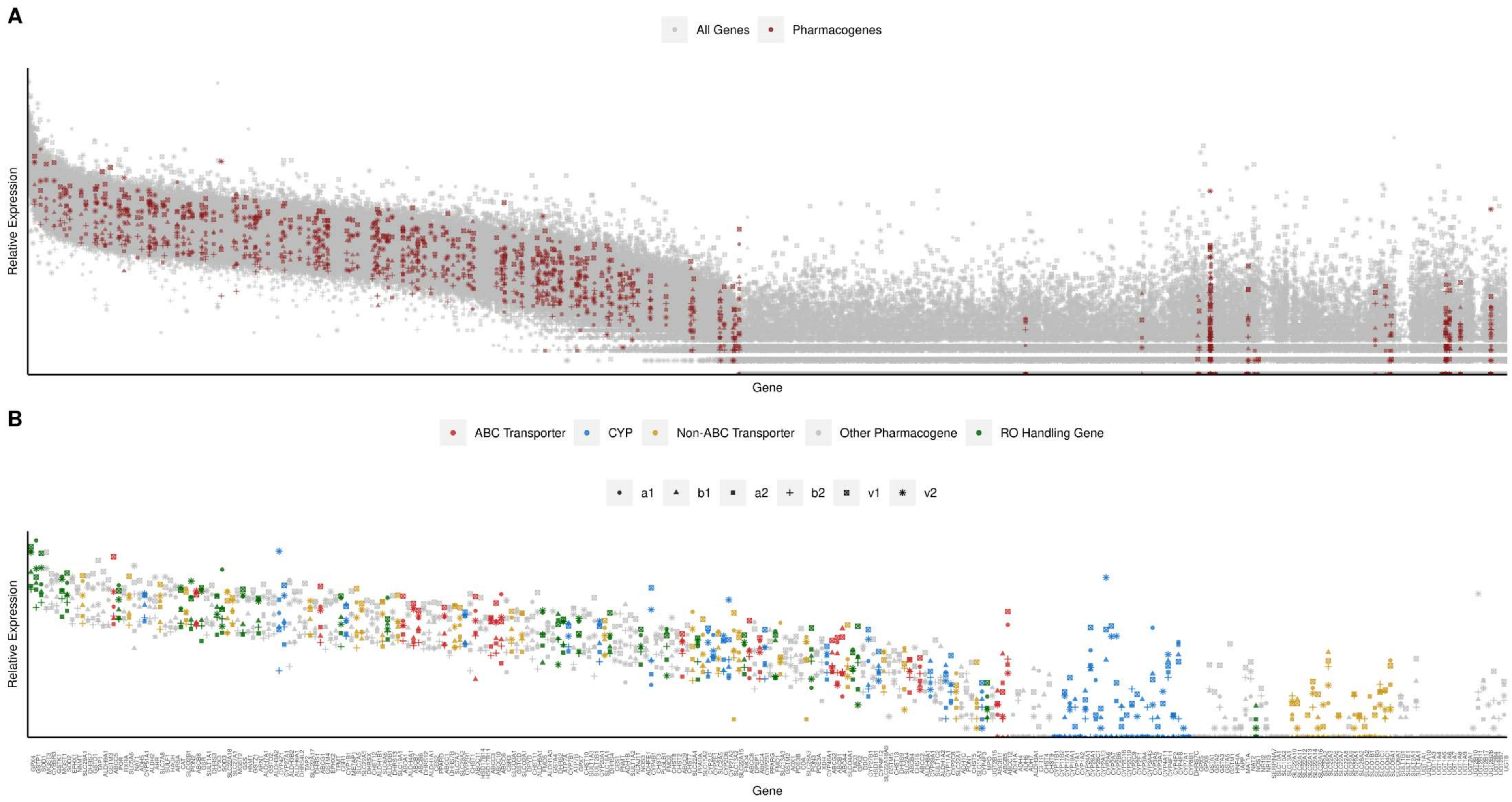

Supplemental Figure S1: Comparison of expression levels of pharmacogenes. Expression is plotted on the y-axis as the log10 of mRNA reads per sample normalized to the total number of spots for that sample for each gene on the x-axis. Order is determined by average of the relative expression in each group. (A) All genes in grey vs. pharmacogenes in red. (B) Pharmacogenes divided by category.

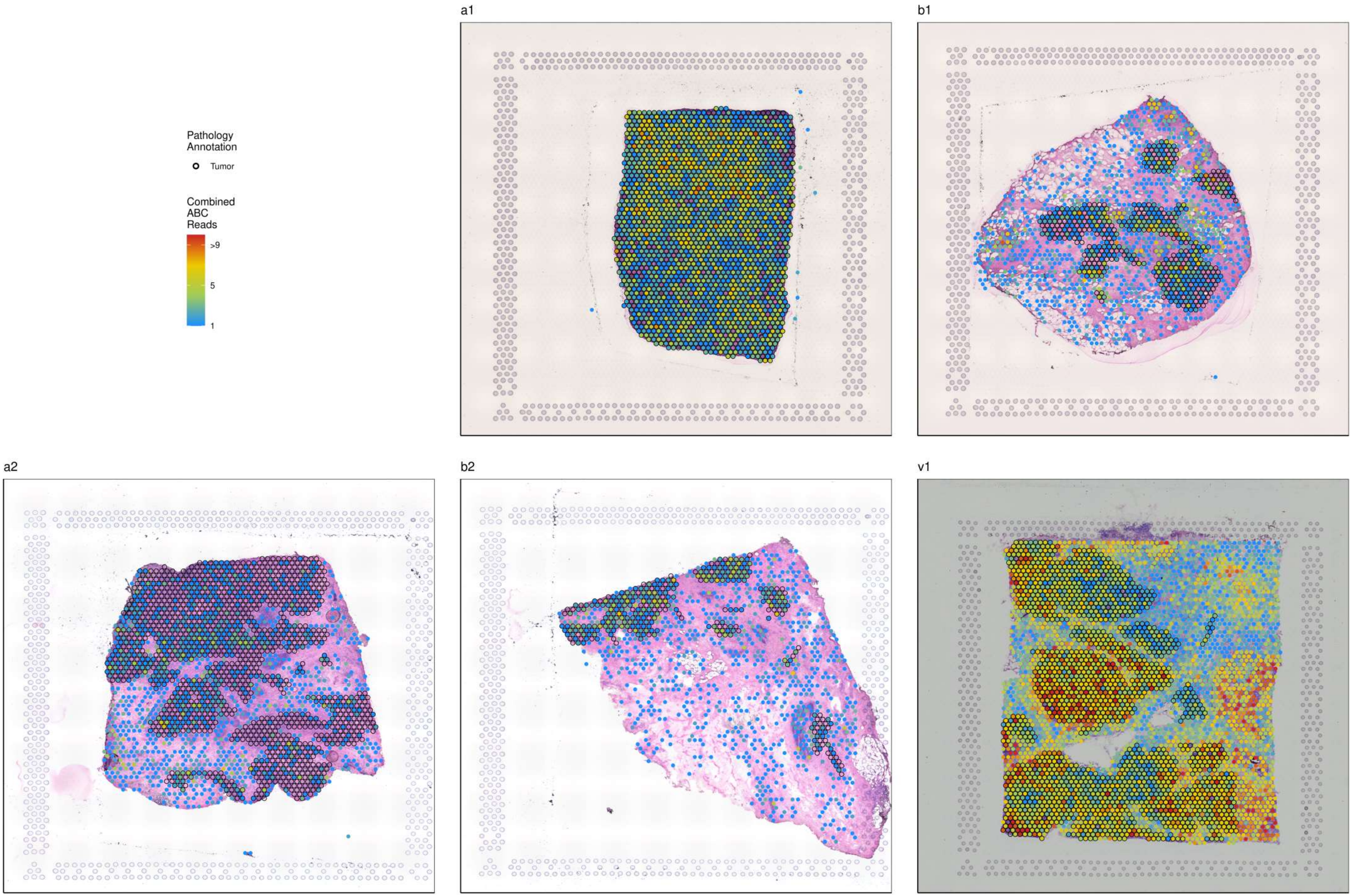

Supplemental Figure S2: Tissue images showing the barcoded dots where combined unique-UMI reads of the ABC transporters are greater than 0. Color intensity represents number of reads, and circles with black outlines denote the regions that were pathologist annotated as tumor.

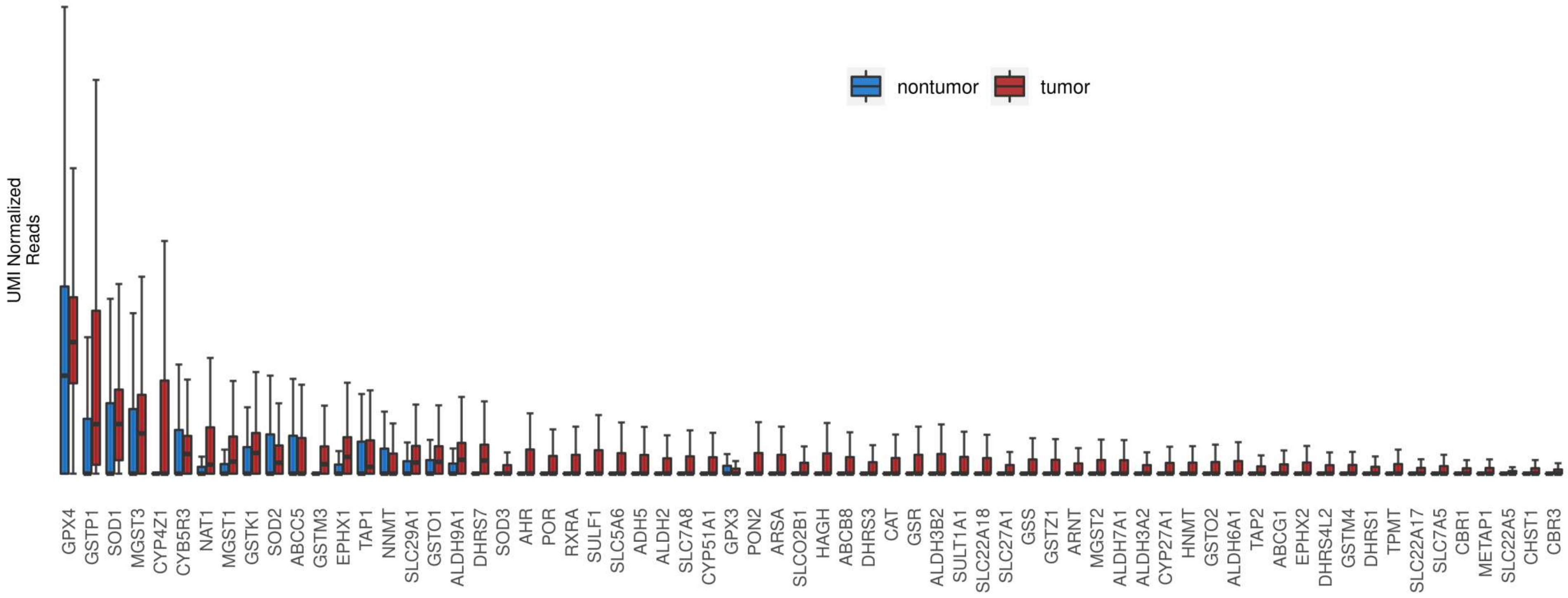

Supplemental Figure S3: Boxplots of UMI-normalized unique-UMI reads for subsets of pharmacogenes with the largest interquartile ranges. Groups divided into tumor (tumor + DCIS+ cellular tumor + desmoplastic tumor) and non-tumor (normal + lymphocytes + stroma), showing the gene subset based on interquartile ranges greater than 0. Y-axis scale is arbitrary, as this figure is intended to show relative differences.
