# Supplementary material for: Quantification of spatial pharmacogene expression heterogeneity in breast tumors
Source: Cancer Rep (Hoboken). 2022 Jul 30;6(1):e1686. doi: 10.1002/cnr2.1686 (PMC9875649; doi:10.1002/cnr2.1686)
Supplement: Supplementary file 3 — Table S1: ROS GO terms and GO IDs. We filtered GO terms using the following key words; “oxidative” (n = 9), “oxygen” (n = 43), and “reactive” (n = 6). We then removed 34 terms that lacked clear ROS function to arrive at 27 unique terms corresponding to 35 ROS pharmacogenes. Table S2: Patient demographics for samples obtained from the Indiana Biobank. Table S3: The 45 most significant GO terms (Bonferroni‐corrected p‐value <.05) overrepresented by the 66 heterogeneously expressed pharmacogenes. Within the PANTHER web tool, we ran statistical overrepresentation tests (Fisher's exact and Bonferroni correction) for the three annotation sets using all human genes in the database as the reference list. [file CNR2-6-e1686-s002.docx]

| Supplemental Table S1. ROS GO terms and GO IDs. We filtered GO terms using the following key words; “oxidative” (n=9), “oxygen” (n=43), and “reactive” (n=6). We then removed 34 terms that lacked clear ROS function to arrive at 27 unique terms corresponding to 35 ROS pharmacogenes. |
| --- |
| **GO term name (GO ID)** |
| Age-dependent response to oxidative stress (GO:0001306) |
| Age-dependent response to reactive oxygen species (GO:0001315) |
| cellular response to oxidative stress (GO:0034599) |
| cholesterol monooxygenase (side-chain-cleaving) activity (GO:0008386) |
| Detection of oxygen (GO:0003032) |
| Intrinsic apoptotic signaling pathway in response to oxidative stress (GO:0008631) |
| Monooxygenase activity (GO:0004497) |
| Negative regulation of calcidiol 1-monooxygenase activity (GO:0010956) |
| Negative regulation of oxidative stress-induced intrinsic apoptotic signaling pathway (GO:1902176) |
| Negative regulation of reactive oxygen species biosynthetic process (GO:1903427) |
| Oxidative demethylation (GO:0070989) |
| Oxidoreductase activity, acting on paired donors, with incorporation or reduction of molecular oxygen (GO:0016705) |
| Oxidoreductase activity, acting on paired donors, with incorporation or reduction of molecular oxygen, NAD(P)H as one donor, and incorporation of one atom of oxygen (GO:0016709) |
| Oxidoreductase activity, acting on paired donors, with incorporation or reduction of molecular oxygen, reduced flavin or flavoprotein as one donor, and incorporation of one atom of oxygen (GO:0016712) |
| Oxygen binding (GO:0019825) |
| Oxygen homeostasis (GO:0032364) |
| Oxygen metabolic process (GO:0072592) |
| Positive regulation of monooxygenase activity (GO:0032770) |
| Positive regulation of monophenol monooxygenase activity (GO:0032773) |
| Positive regulation of oxidative stress-induced intrinsic apoptotic signaling pathway (GO:1902177) |
| Positive regulation of reactive oxygen species metabolic process (GO:2000379) |
| Reactive oxygen species metabolic process (GO:0072593) |
| Regulation of oxidative phosphorylation (GO:0002082) |
| Regulation of reactive oxygen species metabolic process (GO:2000377) |
| Regulation of transcription from RNA polymerase II promoter in response to oxidative stress (GO:0043619) |
| Response to oxidative stress (GO:0006979) |
| Response to reactive oxygen species (GO:0000302) |

Supplemental Table S2: Patient demographics for samples obtained from the Indiana Biobank.

| **Sample name** | **A1** | **B1** | **A2** | **B2** |
| --- | --- | --- | --- | --- |
| **Subject Age (years)** | 52 | 74 | 67 | 52 |
| **Ethnicity** | Not Hispanic or Latino | Not Hispanic or Latino | Not Hispanic or Latino | Not Hispanic or Latino |
| **Race** | White | White | White | White |
| **Gender** | F | F | F | F |
| **BMI** | 31.5 | 20.4 | 25.1 | 29.5 |
| **Weight (kg)** | 78.1 | 52.2 | 61.9 | 72.2 |
| **Height (cm)** | 157.4 | 160 | 157 | 156.5 |
| **Surgery** | Lnd-axillary; mastectomy | Mastectomy | Lnd-axillary; mastectomy | Lnd-axillary; mastectomy/snb |
| **Prior Treatments** | Chemotherapy (taxol and Cytoxan) x6 cycles followed by radiation treatment | Received radiation x10 treatments (nos) to the femur, started on Arimidex x6 weeks, switched to xeloda/ capecitabine x2 cycles | None | None |
| **Pathology Status** | Cancer-primary | Cancer-primary | Cancer-primary | Cancer-primary |
| **Histologic Type** | Infiltrating / invasive ductal carcinoma | Infiltrating / invasive lobular carcinoma | Infiltrating lobular carcinoma | Infiltrating / invasive ductal and lobular carcinoma |
| **Other Specific Site** | Breast, nos | Breast, nos | Upper-inner quadrant of breast | Breast, nos |
| **Side** | Right | Left | Left | Right |
| **Specimen Preparation Type** | Frozen | Frozen | Frozen | Frozen |
| **QC Comments** | 10%T,10%In-Situ,80%stroma | T=65%; necrosis=10% (not inflammatory) | 35%fat,15%stroma,50%T | 95%T; 5% fat |
| **ER/PR; HER2 Status** | ER positive (50%) / PR positive (30%); 1+ | ER positive / PR negative; Negative | ER positive (90%, 3+) / PR positive (99%, 3+); Negative | ER positive / PR positive; 3+ |
| **Grade*** | 7/9 (grade 2) | 7/9 (grade 2) | 6/9 (grade 2) | 6/7 (grade 2) |
| **Grade System*** | Nottingham | SBR | Nottingham | Nottingham |
| **T*** | T4d | T3 | T3 | T2 |
| **N*** | N2a | N1a | N1 | N1 |
| **M*** | Mx | M1 | Mx | Mx |
| **Stage*** | IIIB | IV | Unavailable | Unavailable |
| *Specified final pathology  Abbreviations: BMI (body mass index), QC (quality control), ER (estrogen receptor), PR (progesterone receptor), HER2 (human epidermal growth factor receptor 2), TNM staging (<https://www.cancer.gov/publications/dictionaries/cancer-terms/def/tnm-staging-system>), F (female), Lnd-axillary (lymph node dissection-axillary), NOS (not otherwise specified), SBR (Scarff-Bloom-Richardson) | | | | |

| Supplemental Table 3. The 45 most significant GO terms (Bonferroni-corrected p-value < 0.05) overrepresented by the 66 heterogeneously expressed pharmacogenes. Within the PANTHER web tool, we ran statistical overrepresentation tests (Fisher’s exact and Bonferroni correction) for the three annotation sets using all human genes in the database as the reference list. | |
| --- | --- |
| **GO term name (GO ID)** | **P value** |
| **Biological Process** |  |
| Response to toxic substance (GO:0009636) | 2.29E-25 |
| Detoxification (GO:0098754) | 2.38E-22 |
| Cellular detoxification (GO:1990748) | 1.10E-20 |
| Cellular response to toxic substance (GO:0097237) | 3.53E-20 |
| Cellular oxidant detoxification (GO:0098869) | 3.34E-18 |
| Cellular modified amino acid metabolic process (GO:0006575) | 3.82E-17 |
| Small molecule metabolic process (GO:0044281) | 1.54E-16 |
| Glutathione metabolic process (GO:0006749) | 4.98E-15 |
| Response to xenobiotic stimulus (GO:0009410) | 2.64E-12 |
| Xenobiotic metabolic process (GO:0006805) | 2.67E-11 |
| Cellular response to xenobiotic stimulus (GO:0071466) | 4.64E-11 |
| Oxoacid metabolic process (GO:0043436) | 6.36E-11 |
| Organic acid metabolic process (GO:0006082) | 1.09E-10 |
| Carboxylic acid metabolic process (GO:0019752) | 3.38E-10 |
| Sulfur compound metabolic process (GO:0006790) | 4.31E-10 |
| **Molecular Function** |  |
| Oxidoreductase activity (GO:0016491) | 2.58E-27 |
| Antioxidant activity (GO:0016209) | 4.87E-19 |
| Catalytic activity (GO:0003824) | 1.36E-12 |
| Glutathione transferase activity (GO:0004364) | 1.16E-11 |
| Peroxidase activity (GO:0004601) | 4.46E-11 |
| Oxidoreductase activity, acting on peroxide as acceptor (GO:0016684) | 8.55E-11 |
| Glutathione peroxidase activity (GO:0004602) | 3.16E-10 |
| Transferase activity, transferring alkyl or aryl (other than methyl) groups (GO:0016765) | 7.27E-09 |
| Aldehyde dehydrogenase (NAD+) activity (GO:0004029) | 2.21E-07 |
| Aldehyde dehydrogenase [NAD(P)+] activity (GO:0004030) | 3.03E-07 |
| Oxidoreductase activity, acting on the aldehyde or oxo group of donors, NAD or NADP as acceptor (GO:0016620) | 4.69E-07 |
| Amide transmembrane transporter activity (GO:0042887) | 4.69E-07 |
| Active transmembrane transporter activity (GO:0022804) | 5.28E-07 |
| Glyceraldehyde-3-phosphate dehydrogenase (NAD+) (non-phosphorylating) activity (GO:0043878) | 5.95E-07 |
| Oxidoreductase activity, acting on the aldehyde or oxo group of donors (GO:0016903) | 1.39E-06 |
| **Cellular Component** |  |
| Mitochondrion (GO:0005739) | 1.46E-06 |
| Microbody (GO:0042579) | 9.27E-05 |
| Peroxisome (GO:0005777) | 9.27E-05 |
| Extracellular exosome (GO:0070062) | 1.44E-04 |
| Extracellular vesicle (GO:1903561) | 1.72E-04 |
| Extracellular organelle (GO:0043230) | 1.75E-04 |
| Extracellular membrane-bounded organelle (GO:0065010) | 1.75E-04 |
| Cytoplasm (GO:0005737) | 6.37E-04 |
| TAP complex (GO:0042825) | 1.18E-03 |
| Mitochondrial matrix (GO:0005759) | 1.55E-03 |
| Vesicle (GO:0031982) | 1.56E-03 |
| Apical part of cell (GO:0045177) | 5.00E-03 |
| Apical plasma membrane (GO:0016324) | 1.00E-02 |
| MHC class I peptide loading complex (GO:0042824) | 1.28E-02 |
| Endoplasmic reticulum (GO:0005783) | 1.89E-02 |
